# Revealing the Impacts of Chemical Complexity on Submicrometer Sea Spray Aerosol Morphology

**DOI:** 10.1021/acscentsci.3c00184

**Published:** 2023-05-04

**Authors:** Abigail C. Dommer, Nicholas A. Wauer, Kyle J. Angle, Aakash Davasam, Patiemma Rubio, Man Luo, Clare K. Morris, Kimberly A. Prather, Vicki H. Grassian, Rommie E. Amaro

**Affiliations:** Department of Chemistry and Biochemistry, University of California, San Diego, La Jolla, California 92093, United States

## Abstract

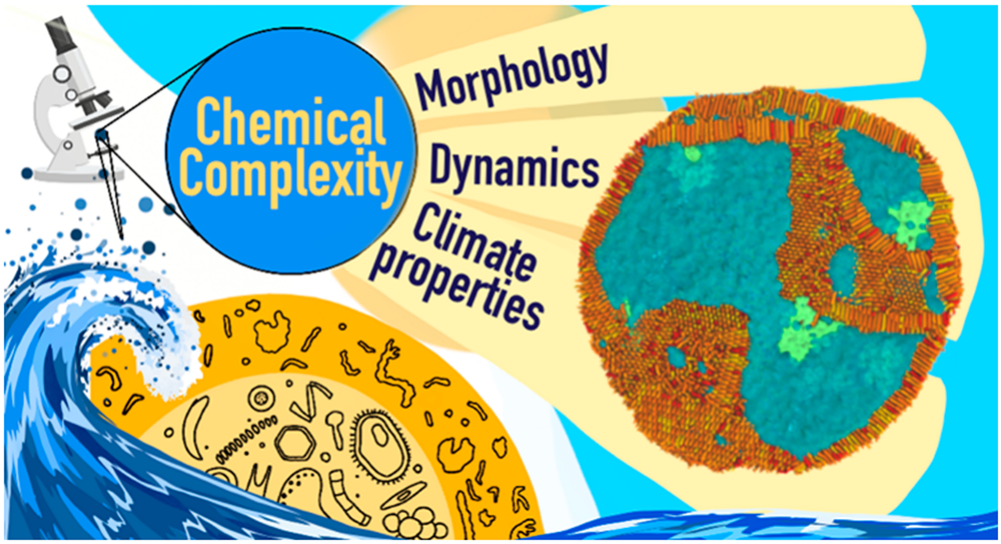

Sea spray aerosol (SSA) ejected through bursting bubbles at the ocean surface is a complex mixture of salts and organic species. Submicrometer SSA particles have long atmospheric lifetimes and play a critical role in the climate system. Composition impacts their ability to form marine clouds, yet their cloud-forming potential is difficult to study due to their small size. Here, we use large-scale molecular dynamics (MD) simulations as a “computational microscope” to provide never-before-seen views of 40 nm model aerosol particles and their molecular morphologies. We investigate how increasing chemical complexity impacts the distribution of organic material throughout individual particles for a range of organic constituents with varying chemical properties. Our simulations show that common organic marine surfactants readily partition between both the surface and interior of the aerosol, indicating that nascent SSA may be more heterogeneous than traditional morphological models suggest. We support our computational observations of SSA surface heterogeneity with Brewster angle microscopy on model interfaces. These observations indicate that increased chemical complexity in submicrometer SSA leads to a reduced surface coverage by marine organics, which may facilitate water uptake in the atmosphere. Our work thus establishes large-scale MD simulations as a novel technique for interrogating aerosols at the single-particle level.

## Abstract 2

Increased chemical complexity in submicrometer sea spray aerosol leads to more heterogeneous morphology and lower surfactant coverage, which may explain the high hygroscopicity of nascent organic-rich SSA.

## Introduction

Atmospheric aerosols impact our climate by nucleating cloud droplets and ice crystals, scattering, absorbing, and reflecting solar radiation, and reacting with or sequestering atmospheric gases (e.g., pollutants). The representation of these aerosols in climate models contributes significant uncertainty to our predictions of global warming or cooling.^1,2^ Sea spray aerosol (SSA) particles released into the atmosphere through bubble bursting at the ocean surface contain a wide range of biological and organic molecules, including proteins, saccharides, alkanes, and even whole or fragmented bacteria and viruses.^3−7^ The organic constituents of SSA are derived from marine microbial processes, which in turn influence the number concentrations of particles that can later form cloud droplets.^8−13^ While supermicrometer aerosol particles readily nucleate clouds due to their size, the cloud-forming potential of submicrometer SSA is controlled by the chemical complexity of the organic components and their morphologies.^14,15^

Fine SSA in particular, with diameters of <200 nm, contain nearly 100% organic material by mass, yet observations indicate that these aerosols have hygroscopicity parameters approaching those of pure salt particles, i.e., they take up water as if they were salty.^8,14,16,17^ It has been demonstrated for simple aerosol systems that morphology alone can directly influence hygroscopicity. Altaf et al. showed that homogeneously mixed organic aerosols take up water less readily than those of the same chemical compositions exhibiting complete phase separation.^18,19^ The discrepancy between the predicted and the observed water uptake of fine SSA is likely explained by morphology, but their small sizes and broad chemical complexity make them particularly challenging to study using single-particle analysis methods. Nevertheless, unraveling the relationship between chemical complexity and morphology will give us a better understanding of SSA climate impacts and enable better parametrizations of aerosol–cloud interactions in atmospheric models.

Aerosol morphology is a critical driver of many atmospheric properties; particle surfaces mediate interactions with gases and sunlight,^20−24^ while the organization of the aerosol interior regulates the absorption and diffusion of small molecules which can control the phase state and water uptake.^25−27^ The current understanding of aerosol morphology is derived from experimental observation. Organic aerosols adopt a variety of morphologies, including the common core–shell morphology,^15,26,28−32^ in which organic materials phase separate to the aerosol surface to form a thick coating around a salty aqueous core. Other phase-separated morphologies besides core–shell have been observed for model systems (Figure S1) and depend on the particle size,^19,33,34^ O:C ratio of the organic species, and/or organic: inorganic mass ratio.^28,35−43^ However, observations using single-particle analysis methods can be compromised by a variety of factors including the aerosol generation mechanism, sample storage, and analysis method.^44−49^ Additionally, all microscopy-based techniques such as atomic force microscopy (AFM), scanning electron microscopy (SEM), and cryogenic transmission electron microscopy (cryo-TEM), while valuable, require the deposition of aerosols onto a substrate which alters the particle shape and surface area,^31,50^ compromising the structural integrity of the particle. Techniques such as microfluidics and aerosol optical tweezers (AOT) are similarly limited in their size resolution and aerosol generation methods and, for AOT, the ability to investigate nonspherical particles within a single laser beam trap.^51,52^

Molecular dynamics (MD) simulations (a.k.a. computational microscopy) provide an effective alternative to conventional experimental techniques and have been used to interrogate a variety of properties of nanoscale aerosols, including water condensation,^53−59^ atmospheric gas uptake,^60^ coalescence,^61^ and the phase state.^29,55,57,58,62−66^ A majority of these MD studies are related to natural or anthropogenic volatile organic carbon and investigate small aerosol clusters containing low-molecular-weight mono- and dicarboxylic acids.^30,43,57,62,67−69^ Studies of marine aerosols to date have approximated SSA by combining medium-chain fatty acids or free amino acids with saltwater clusters.^56,70−72^

A central limitation to all MD studies is balancing system size and complexity with simulation length; the simulation of large, complex aerosols for statistically significant time scales is limited by the availability of large-scale (leadership-class) computational resources and the scalability of the MD codes. Thus, aerosol models studied have largely contained simple binary or ternary mixtures on the order of <100 Å in diameter and are simulated for a duration on the order of <100 ns,^29,30,63,66,68,73^ which may limit statistically rigorous sampling. Nevertheless, simulations have supported experimental evidence that insoluble organics fully phase separate into a core–shell or partially engulfed morphology,^74^ albeit with short time scales, small system sizes, and a marked lack of organic complexity.

In the present work, we use large-scale classical all-atom MD simulations to bridge the gap between limited experimental resolution and small-scale MD studies by (1) extending the aerosol size to a 40 nm diameter, a 4-fold increase in diameter—or 64-fold increase in volume—in comparison to previous all-atom MD simulations and (2) increasing the chemical complexity of both the organic and inorganic phases to better represent that of nascent sea spray. We simulated three different 40-nm-diameter aerosol systems for 500–1000 ns, each in triplicate. For each set of replicates, we increased the chemical complexity of the organic phase in increments, beginning with a simple fatty acid (FA) and protein mixture (system A) and then adding perturbations of highly charged lipopolysaccharides and cation diversity (system B), followed by the addition of neutral glucose mono- or oligosaccharides (system C). We evaluated the particle shape and size and tracked the partitioning of organic material. We then compared our simulation results to Brewster angle microscopy (BAM) imaging of model surfactant interfaces. Our work gives insights into how the particle phase and morphology are impacted by chemical diversity and how that in turn may impact climate-relevant properties of submicrometer sea spray. Our simulations also establish computational microscopy as a powerful method to explore aerosols at the single-particle level, augmenting and extending current experimental capabilities.

## Methods

### Experimental Design

To evaluate the impacts of chemical complexity on submicrometer SSA morphology, we used all-atom molecular dynamics simulations to study whole aerosol model systems and supported our results with experimental imaging tools. Our models were designed to address various technical and practical limitations. First, the CHARMM36 force field^75−77^ was used for all simulations, and care was taken to ensure that only molecules that had been sufficiently parametrized and characterized with the force field were investigated. All ions and their concentrations were kept within the force field accuracy limitations. The latest NBFixes (2019) to the CHARMM36 parameter set for Na^+^ and Ca^2+^ were applied. Second, 30–50 nm diameter aerosols are the most difficult to study experimentally due to their small size yet correspond to computationally large systems containing many millions of atoms, which require leadership-class computing facilities. We were thus limited by computational resources and had to compromise on complexity and size such that the simulation length scales would ensure proper sampling and still give statistically rigorous results. Finally, the largest limitation was capturing the vast chemical complexity of nascent SSA. There is broad agreement on the major classes of organic compounds, but narrowing our selection of molecular components to include the most chemically relevant species was nontrivial. We ultimately chose to build three 40-nm-diameter aerosol systems to be run in triplicate (9 total simulations), with each system representing a step up in chemical complexity; i.e., with each new set of models, we added a new molecular component to the organic mass fraction while keeping the organic mass fraction as a whole constant. A more detailed discussion of the force field parameters, inclusion or omission of particular molecular species, and experimental validation are outlined in the Supporting Information.

**Figure 1:**
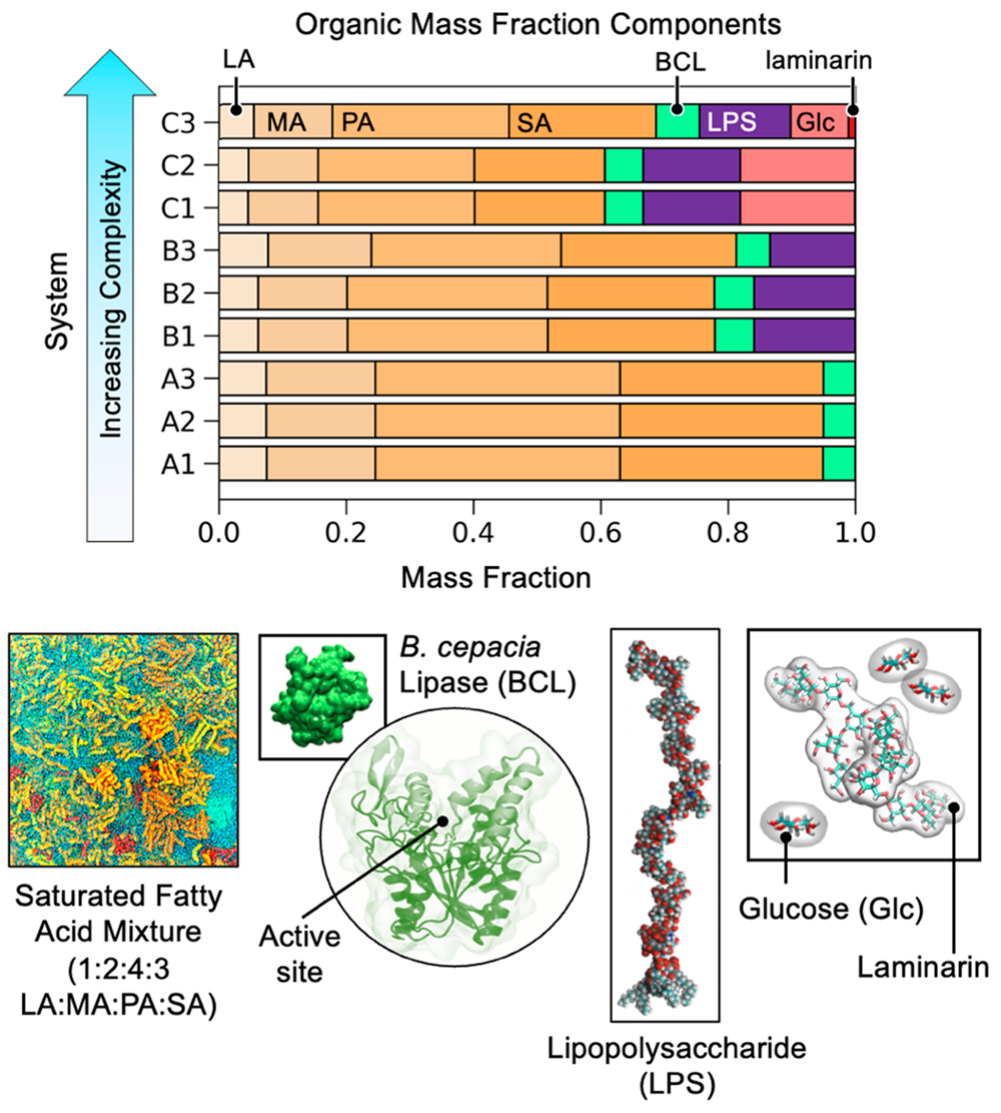
Top: bar chart of organic fraction components by mass for each replicate of each system. Fatty acid abbreviations: lauric acid (LA), myristic acid (MA), palmitic acid (PA) and stearic acid (SA). Bottom: different components are illustrated, and abbreviations for other components are provided.

The chemical components of the SSA model systems were selected based on the most recent molecular analyses of nascent submicrometer SSA. At 70% relative humidity, for SSA < 200 nm in diameter, it is estimated that the mass ratio of inorganic:organic:water is approximately 0.09:0.40:0.51, and thus our models were constructed with these approximate ratios.^78^ It is worth noting that only an estimated 25% of the total chemical species found in SSA has been fully characterized. There is, however, a consensus on the classes of molecules observed, namely, fatty acids, proteins, and saccharides, though their relative abundances in submicrometer SSA vary with ocean productivity, geographic region, season, and temperature.^5,6,10,79,80^ Fatty acids (FAs) comprise 2–80% of the observed organic species by mass, with chain lengths varying from 12 to 18 carbons for the most abundant subset.^6,7^ Our simplest system (system A, Figure 1) is composed of an organic fraction containing only saturated FAs in a 1:2:4:3 ratio of lauric acid (LA):myristic acid (MA):palmitic acid (PA):stearic acid (SA), with chain lengths of 12, 14, 16, and 18 carbons, respectively.^7^ The mass fraction of proteinaceous material in SSA is also highly variable between individual particles. We chose to initialize our simple system with a number of *Burkholderia cepacia* lipases (BCLs). BCL is a robust enzyme found in SSA that has, notably, been observed to retain some activity after aerosolization.^4,81,82^ This enzyme was also selected because of its well-characterized structural, biological, and enzymatic properties as well as the ease with which collaborators can incorporate it into subsequent laboratory studies.^83−85^ The number of lipases remains constant throughout our study at a mass fraction of approximately 3%. Finally, we initialized our simplest system with 0.4 M NaCl to reflect the concentration of seawater and nascent SSA.

To understand the specific impacts of chemical complexity on submicrometer SSA, we chose to increase both the cation complexity as well as the complexity of the organic species. In the next two steps (systems B and C), the aqueous phase contained approximately 110 mM Mg^2+^, 25 mM Ca^2+^, and 20 mM K^+^, in addition to the existing NaCl concentration. When considering the components of the organic fraction, we focused on systems that had been studied before both computationally and experimentally so that our simulations could ultimately be validated by laboratory experiments. For the first step (system B, Figure 1), we added lipopolysaccharides (LPS), which are components of bacterial cell membranes that are often found in SSA due to cellular breakdown. We expected that this addition would be a valuable perturbation to the simple system in two major ways. First, it has been observed that LPS-containing particles readily take up and react with nitric acid in the atmosphere and are thus chemically significant.^25,86^ Second, based on previous studies, we expected that the interaction between LPS and divalent cations such as Ca^2+^ would cause a meaningful phase change within the particle that could be explored computationally.^25,87−92^

Finally, it is estimated that at least 2–10% of the organic carbon mass in submicrometer SSA is made up of poly- and oligosaccharides as well as free saccharides, the most abundant monomer of which is glucose.^78,93,94^ Based on this data, for the final system (system C, Figure 1), we added a combination of free glucose and laminarin, a neutral glucose oligosaccharide. Recently, Richards et al. demonstrated that some monosaccharides in sufficiently high concentrations and in the presence of divalent cations—typical conditions within atmospheric aerosols—form supramolecular ion-bridging interactions that can induce phase transitions to a rigid gel.^27^ This unique phase behavior had been observed for macromolecular systems (such as those containing LPS described above) but not yet with small molecules. This final step up in complexity allows us to explore the impacts of neutral saccharides within the overall mixture and computationally explore any noncovalent bridging interactions that could lead to phase changes. Figure 1 outlines all three systems and the compositions of their respective organic mass fractions.

We note that although the term “replicate” is used here, the model particles within each category are not strictly identical. Each particle is initiated with randomly packed components to reach a target mass percentage, with minor variations in composition between individual models. This approach enables our *in silico* models to reflect the heterogeneity of naturally occurring SSA.

The different chemical compositions we explore in this work are approximations of realistic complexity based on laboratory observations. Given the enormous molecular diversity within SSA, our approach was to start with a simple baseline and test small perturbations to understand the unique impacts of each contribution, which also gives us the opportunity to explore and extract previously unknown and untested intermolecular interactions in atomistic detail. Additionally, the work described in this article represents primary, rather than aged, SSA. The Supporting Information provides further details regarding our experimental design.

### Constructing Sea Spray Aerosol Models

All FA and glucose structures are readily available and parametrized in the CHARMM36m force field. The *E. coli* LPS structure included here was generated using CHARMM-GUI’s LPS Modeler^95^ with type 1 lipid A, an R1 core, and eight O11-antigen repeats. Additional details for this structure can be found in ref (25). The laminarin oligosaccharide model was constructed using the CHARMM-GUI glycan modeler.^96,97^ Laminarin naturally contains a variety of molecular weights and branching ratios, a complexity that is nearly impossible to recreate computationally. However, given the small size of our systems and the high variability across single particles, we opted to create one laminarin structure to represent neutral, branched oligosaccharides. Thus, a polymeric structure of a molecular weight between that of free glucose and the LPS model was generated with 15 glucose monomers, linked together with β(1 → 3) linkages, with branches incorporated at monomers 3 and 9 by β(1 → 6) linkages (Figure S2). The structure of BCL (PDB ID: 3LIP)^98^ was prepared for a pH 5 environment using NAMD *Psfgen*.^99^ The number of water molecules was calculated based on an estimated relative humidity for the particles of 70–80%, amounting to ∼50% water by mass.^78^

All molecular structures were randomly packed into 16 geometrically distinct segments (to later combine into a sphere) using PACKMOL^100^ in 3 successive rounds of packing. The most difficult structures to place (macromolecular structures BCL, laminarin, and LPS) were packed first, followed by small molecules including FAs and glucose, and finally ions and TIP3P^101,102^ water. Small fluctuations in the random seed in the PACKMOL packing algorithm introduced subtle differences in the number and locations of each ingredient placed for each of the packed segments. Thus, the initial molecular configurations between replicates of the same system contain small random variations and reflect variations one might find between particles when doing single-particle analysis.

### All-Atom Molecular Dynamics Simulations

The authors received an allocation through the NSF LRAC program to run dynamics on the UIUC Blue Waters and TACC Frontera supercomputing facilities. Memory-optimized NAMD 2.14, which has excellent scaling for very large systems across thousands of nodes,^99,103−105^ was used to run all all-atom explicit-solvent molecular dynamics simulations; a NAMD efficiency scaling plot across UIUC Blue Waters and TACC Frontera is included in Figure S3 for the curious reader. Each 40 nm aerosol was placed in an empty periodic box cell approximately 55 nm on a side. The authors note that, due to the large size of the SSA model, small deformations in the aerosol as it evolves could cause it to interact with itself across a PBC boundary if the box is not sufficiently large. We found that a 75 Å buffer on all sides of the aerosol particle prevented self-interaction.

Each system was energy minimized, heated, and equilibrated in multiple steps. Conjugate-gradient energy minimizations were performed with FA headgroups harmonically restrained first at 100 kcal/mol Å^2^ and then at 10 kcal/mol Å^2^ for 15 000 cycles each. The systems were heated from 23 to 298.15 K in increments of 25 K for 100 ps each at a 2 fs time step. Finally, the systems were equilibrated at 298.15 K by slowly releasing harmonic restraints on the lipid headgroups over 500 ps. Production runs were carried out with an NVT ensemble over 500 ns for each system and extended to 1 μs in total for the A1 and C3 replicates. For all procedures, particle-mesh Ewald^106^ electrostatics were employed for long-range electrostatic interactions; nonbonded van der Waals interactions and short-range electrostatics were calculated with a 12 Å cutoff. The SHAKE^107^ algorithm was used to fix hydrogen bond lengths, and a Langevin thermostat with a damping coefficient of 5/ps was applied to maintain temperature control at 298.15 K. Table S1 details the atom counts for each system as well as the total time simulated for each replicate.

### Brewster Angle Microscopy

For Brewster angle microscopy experiments, a MicroBAM (KSV NIMA) was used in conjunction with a Langmuir Trough (KSV NIMA LB, S/N AAA100505). All solutions were prepared in Milli-Q water with a resistivity of >18.1 MΩ. NaCl solutions were prepared using sodium chloride (Fisher) that was baked overnight at 200 °C to evaporate organic contaminants. Lipase solutions were prepared at a concentration of 16.5 mg of lipase/100 mL of 0.4 M NaCl solution using lipase from *Pseudomonas cepacia* (BCL) (≥30 U/mg, Sigma-Aldrich). LPS solutions were prepared at a concentration of 1 mg of LPS/100 mL of 0.4 M NaCl using lipopolysaccharides from *Escherichia coli* (O111:B4, Sigma-Aldrich). Hereafter, 0.4 M NaCl solutions containing either LPS, lipase, or no additional component (for control experiments) will be referred to as the subphase. Monolayers were prepared using a 1:2:4:3 ratio of LA:MA:PA:SA. Palmitic acid (>99%, Sigma-Aldrich), stearic acid (>98.5%, Sigma-Aldrich), myristic acid (>99.5%, Sigma-Aldrich), and lauric acid (99%, ACROS) were individually prepared as 1 mg/mL chloroform (>99.9%, Fisher Scientific gold label) solutions. These four individual solutions were then mixed by volume according to the 1:2:4:3 ratio.

To begin an experiment, a paper Wilhelmy plate was first soaked in the subphase for 30 min. An ethanol-cleaned, fully dried black glass plate was placed in the trough to prevent unwanted radiation scattering from the BAM laser. The trough was then filled with ca. 52 mL of subphase, and the plate was transferred to the balance hook and lowered to the first point of contact with the subphase in the trough. At this point, the balance was set to zero. A microsyringe was then used to spread a monolayer on the subphase using ca. 20 μL dropwise. This was accomplished by depressing the plunger just enough to create a droplet and then touching this droplet to the subphase without contaminating the syringe. Droplets were deposited in different locations to spread the monolayer effectively. After a sufficient monolayer was spread, the chloroform was allowed to evaporate for 20 min. The BAM head was positioned over the trough, and initial images were collected. Compression with a single barrier was then carried out at a rate of 10 mm/min, and images were frequently taken to observe morphology changes in the monolayer, with the surface pressure balance readings manually recorded with each image, up to ca. 30 mN/m. Images were then analyzed, and features unique to LPS or lipase subphases that did not occur for NaCl-only control experiments are presented in this report.

## Results and Discussion

MD simulations were performed on the nine systems described above with the chemical compositions given by Figure 1. The chemical ingredients were distributed randomly during packing to allow the components to self-assemble without introducing bias into the system (SI Movie M1). The models were evaluated over the course of the production runs to track the evolution of their shape and the aggregation of FA over time. Equilibrium was assumed to be reached after approximately 100 ns of production, at which point the particle shapes stabilized and the majority of the FAs were organized into clusters. Their morphologies at the final time steps are given in Figure S4.

### Shape Evolution and Dynamics

**Figure 2:**
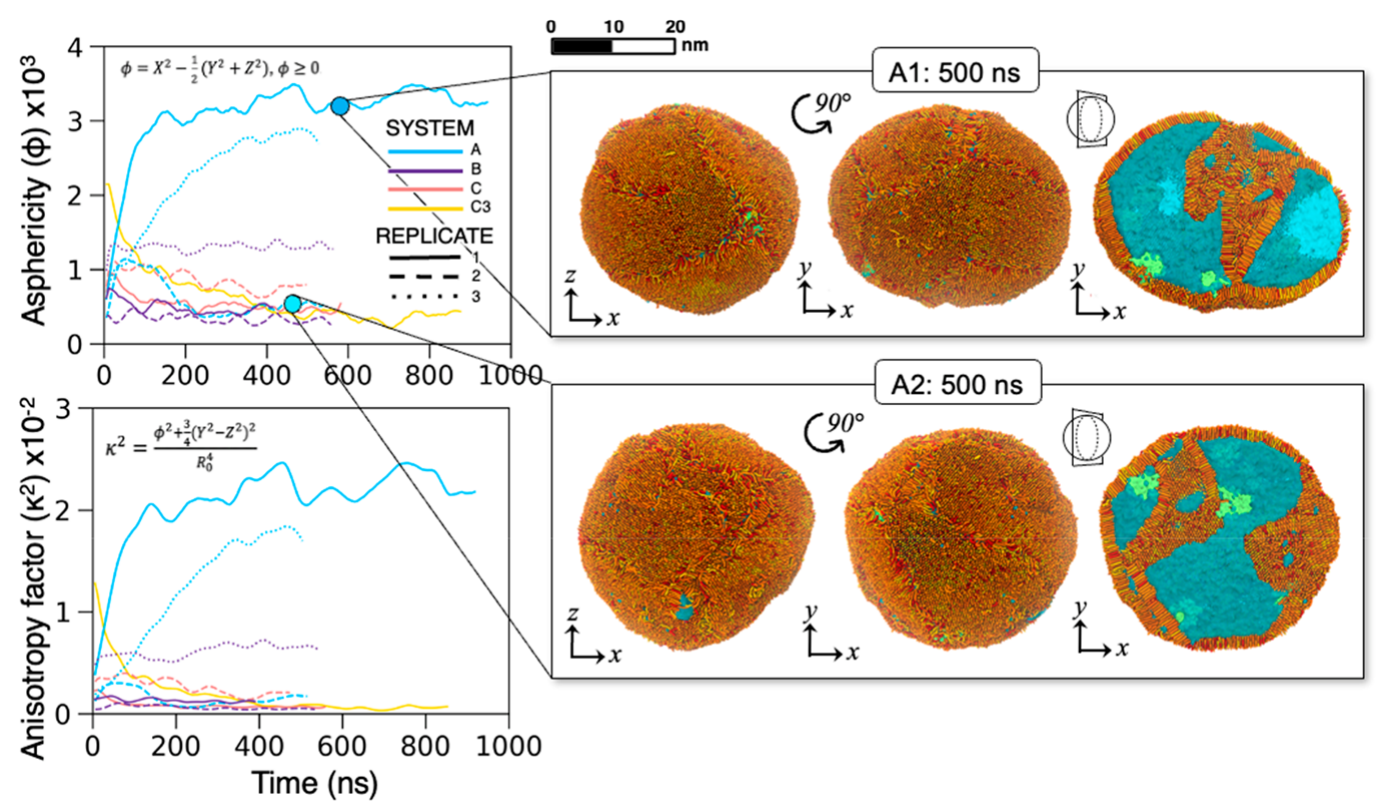
Evolution of aerosol shape over time, designated by the asphericity factor (top left) and relative shape anisotropy factor (bottom left). Right: the two shapes adopted by SSA (ellipsoidal and spherical) are represented here by the simplest system replicates A1 and A2. MD snapshots of these replicates corresponding to the *xy* and *xz* planes at 500 ns are provided for visual shape comparison. Lipids are colored orange, red, and yellow; BCL is colored green; and water is colored blue.

To track the evolution of aerosol shape, we quantified the degree of asphericity, ϕ, and the relative shape anisotropy parameter, κ^2^, which can be derived from the radius of gyration tensors. As the aerosols evolved, their initial spherical shape often deformed into an ellipsoid which can be characterized using the asphericity term. This term is zero when the particle is a perfect sphere and increases as the shape evolves away from sphericity. In a complementary analysis, κ^2^ was used to describe the symmetry of the particle. For rod-like symmetry, κ^2^ approaches 1, where all atoms lie along a line, while a value of 0 indicates that the particle has a higher degree of symmetry, such as that of a perfect tetrahedron or sphere. The evolution of ϕ and κ^2^ for each replicate of each aerosol system is plotted over time in Figure 2. This analysis of aerosol shape was based on that developed by Karadima et al.^29,30,108^ A detailed description of the calculations and derivations used here is given in the Supporting Information.

Figure 2 indicates that the largest deviations from sphericity occur with the evolution of the A systems, which contain organic fractions composed of only FAs. A1 and A3 specifically show the largest changes in symmetry, corresponding to the most dramatic changes in the κ^2^ parameter. We provide MD snapshots from the 500 ns time point for replicates A1 and A2 to illustrate. A1 evolves into an ellipsoidal shape within the first 100 ns of simulation time. A2 initially approaches and hovers around ϕ = 1000 before falling to ϕ = 500 at 200 ns, which corresponds to a more spherical and symmetric structure than A1 and more closely resembles the shape of the other systems.

The more dramatic evolutions in particle shape appear to correspond to the higher FA content relative to that in the other systems. FAs, particularly protonated FAs, have high hydrophobicity and favor the formation of highly ordered structures on a rapid time scale. This self-assembly has been explored experimentally on medium- and long-chain FAs in aqueous solutions.^109^ Once the surface saturates with a monolayer, FAs aggregate in bulk, forming micelles and lamellar vesicles of tunable size based on the fatty acid chain length, temperature, pH, and salt concentration.^109−119^ Indeed, vesicle formation within intact supermicrometer sea spray has been observed previously by Patterson et al. via cryogenic transmission electron microscopy.^44^ However, submicrometer aerosols, shown by our simulations, do not have sufficient volume for micelle or vesicle formation. Rather, the excess lipids—those not occupying space at the surface—aggregate into amorphous oil droplets that either adsorb to the monolayer or are freely suspended in the aqueous phase. The aggregates that form in the present simulations often take the form of bilayers and/or lipid agglomerates. For A1 and A3, the aggregate is a bilayer that bisects the aerosol to form an ellipsoidal shape; in A2, the aggregates are double bilayers that adhere to either side of the particle, maintaining the observed spherical shape (Figure 2).

To understand how the organic material is distributed between the aerosol surface and interior, we evaluated the aggregation dynamics of FAs using a clustering analysis. Clusters were determined by the molecular position and the vector formed between the head and tail carbon atoms with respect to the surface normal using the DBSCAN clustering method. This method was selected because it does not require a predetermined number of clusters and allows for individual lipids to be assigned to an “unclustered” group. The carbon vector was selected as a clustering parameter to characterize the formation of monolayers in which all lipids obtain a similar directional orientation and tilt; that is, the hydrophobic tails align and the carboxylic headgroups face the aqueous phase.

**Figure 3:**
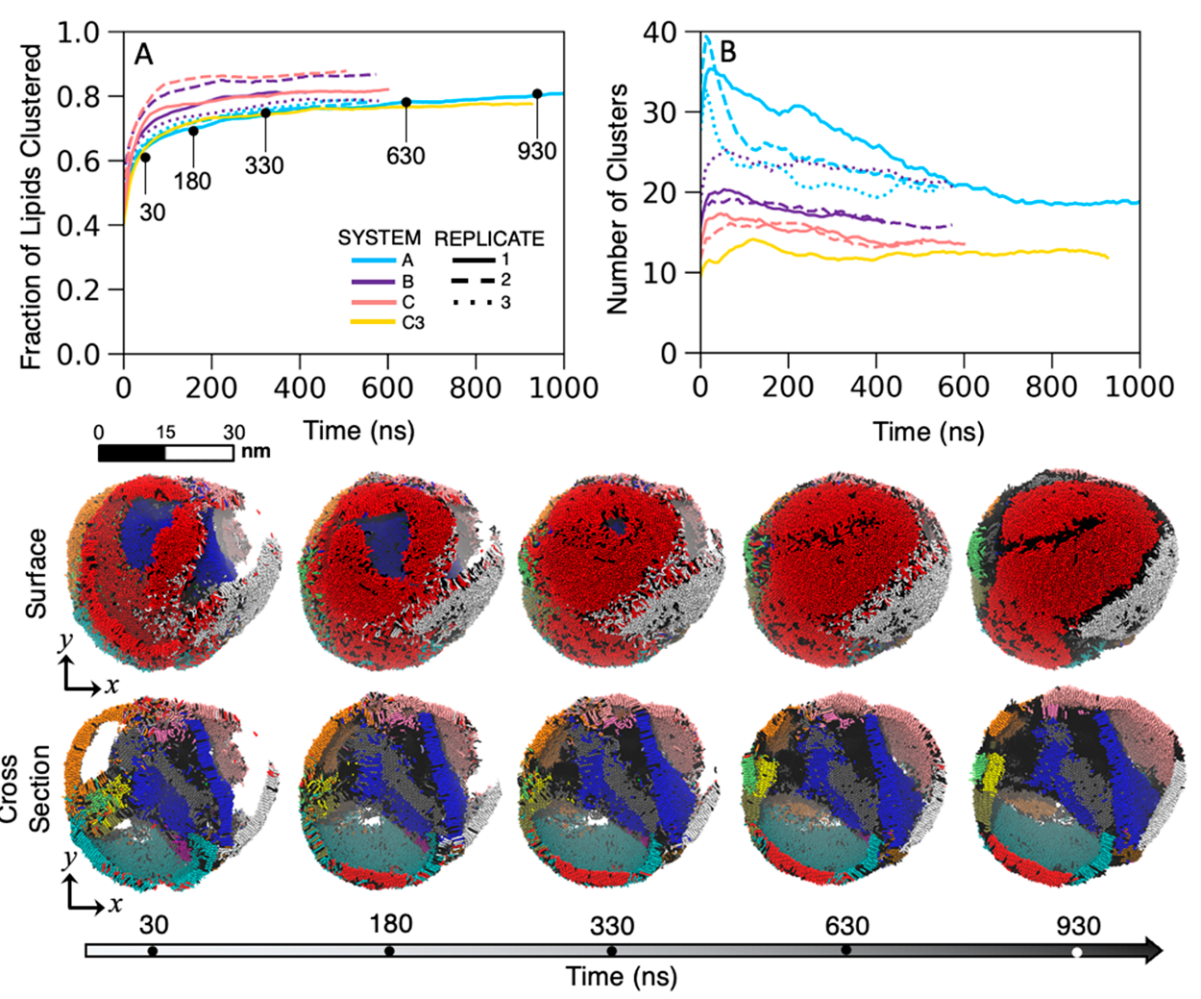
Lipid clustering analysis. DBSCAN clustering reveals the kinetics of lipid aggregation over the course of the simulation. (A) Fraction of clustered lipids over time, with time points labeled in ns. (B) Total number of clusters evolved over time. In the lower panel, we provide snapshots from MD simulations of the A1 replicate at the given time point with unique clusters colored by ID. Only the lipids are visualized.

The results of this analysis show that FAs readily aggregate rapidly into long-lived clusters in under 100 ns, with over 60% of the total lipids clustered in the first 30 ns. Figure 3 shows the fraction of total lipids clustered as well as the total number of clusters calculated over the duration of the simulations. Snapshots from the MD simulations for A1 are provided with each cluster colored individually. Upon visual inspection, the first time point (30 ns) already shows lipids organizing into their final clusters, with latter time steps illustrating increased molecular alignment, greater surface coverage, and fewer unclustered patches. By the final time steps, the surface is nearly saturated (SI Movie M2), and the lipids are aligned into rafts, or patches of similarly aligned FAs at the particle surface. Two major rafts are visible in the snapshots in Figure 3, colored in red and gray, with unclustered individual FAs in black between the clusters. The individual rafts are distinct from one another by the tilt angle and form a patchwork pattern over the surface. (Note that we use the term “raft” here to describe a surfactant monolayer patch in which the lipids aggregate at the air/saltwater interface and adopt a similar tilt angle and orientation. This term is not to be confused with the phenomenon occurring in cellular membranes)

### Morphology

**Figure 4:**
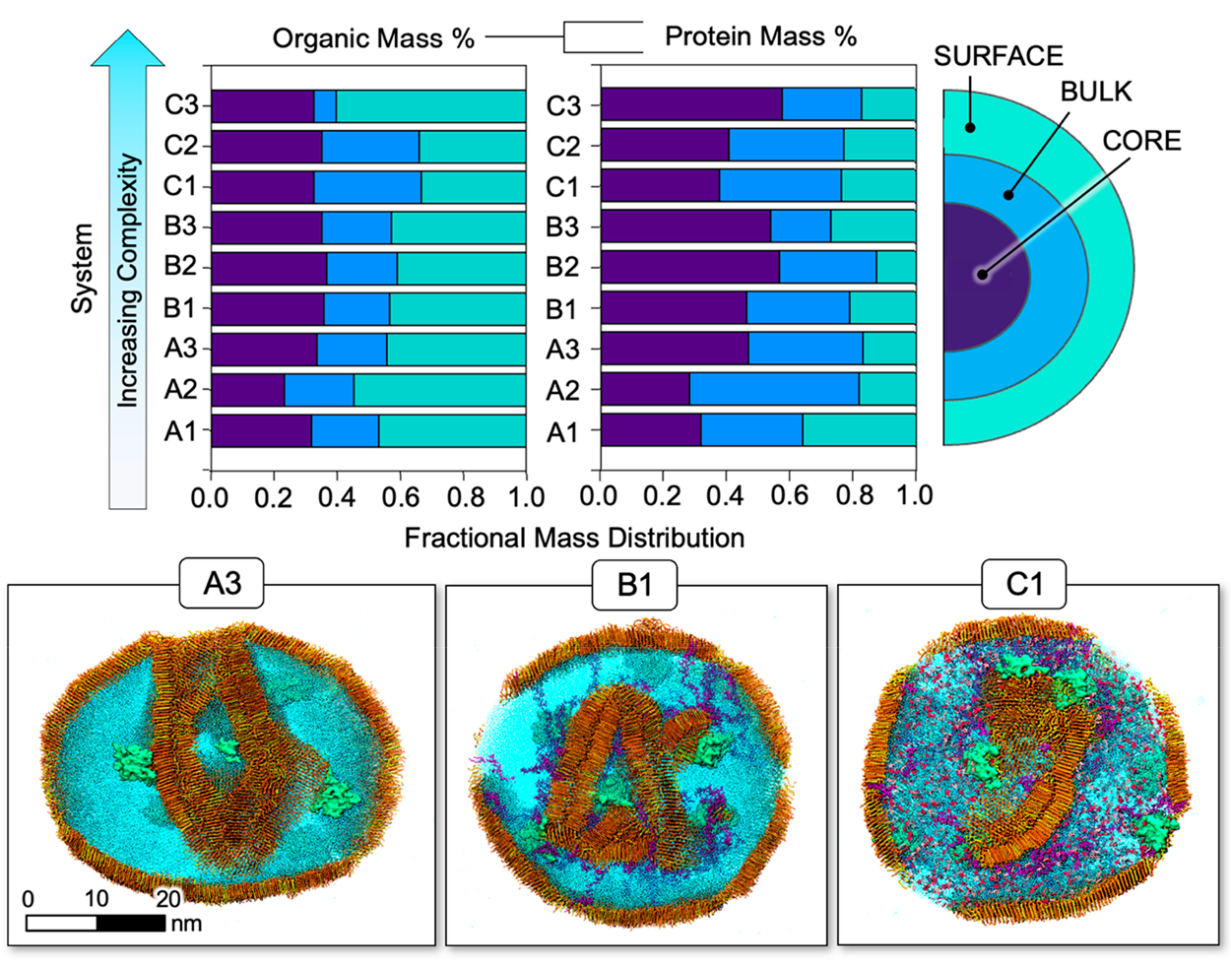
Distribution of organic material throughout the particle. Top: distribution of organic material (left) and protein-only material (right) by mass percentage throughout the surface, bulk, and core regions. (See the illustration to the right for region labels.) Bottom: selected snapshots representing cross sections from A, B, and C replicates at their final time steps. Organic material includes all fatty acids, BCL, LPS, glucose, and laminarin. Protein-only material accounts for only the BCL mass.

To quantitatively evaluate the distribution of organic material throughout each aerosol, we subdivided each particle into concentric ellipsoids of equal volume, following the methods developed and described by Karadima et al.^30^ For the last snapshot of each simulation, we applied a convex hull method to approximate the bounding ellipsoid and then found a best-fit ellipsoidal mesh to describe the surface of the aerosol (see the Supporting Information for details). Using the equations for the best-fit ellipsoidal mesh, we then calculated the equations representing concentric ellipsoidal shells encompassing equal volumes. Using atom selections in VMD,^120^ we extracted the atoms located in each region defined as the surface, the bulk, or the core and calculated their mass percentages. We then plotted the distribution of material by mass throughout the three regions (Figure 4).

This analysis shows that the organic material is largely distributed to either the surface or the core (SI Movies M3 and M4). Additionally, there is little correlation between the distribution of organic material with the addition of chemical complexity. Since the majority of the organic material in our simulations is amphiphilic or partially water-soluble in character, the particles do not exhibit complete aqueous/organic phase separation. Both FAs and LPS contain hydrophobic tails and polar headgroups and can thus participate in monolayer, micellar, and bilayer aggregation. Glucose, due to its small size and hydroxy groups, is highly soluble and will remain in solution, integrating into the hydrogen bond network of the aqueous phase. Bulky, branched laminarin, although less soluble than glucose, still contains polar hydroxyl groups that lend it partial solubility.

The macromolecular organic components explored in this work, namely, LPS, laminarin, and BCL, readily aggregate into inclusions in the aerosol center and are stabilized by FA clusters. This finding supports the core–shell morphology to a limited extent; in addition to saturating the surface, organics, and specifically BCL, also accumulate in the center of the particle. Mael et al. recently observed this phenomenon during water uptake experiments: neither complete phase separation nor full dissolution occurs in the case of some organics, leading to the formation of inclusions.^121^ Additionally, Huang et al. observed that organic aerosols with an organic component containing an O:C ratio <0.8 exhibited phase coexistence.^32^ The O:C ratios of our SSA models align well with this observation (Table S3).

**Figure 5:**
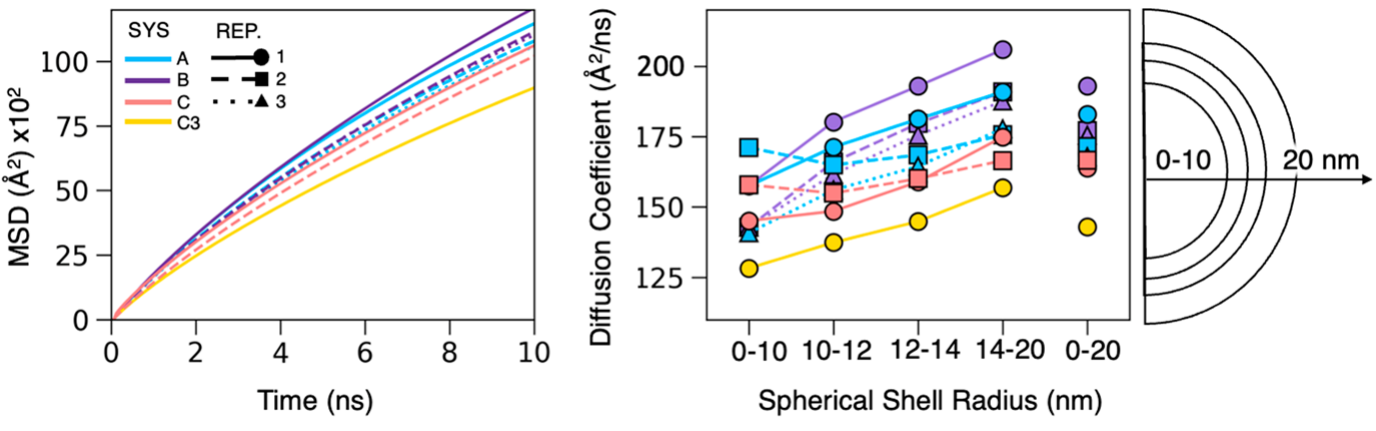
Diffusion analysis for water transport throughout each model system over the final 50 ns of each trajectory. Left: mean squared deviation (MSD) for all water molecules averaged over five 10 ns time slices indicating a slight correlation between increasing chemical complexity and slowed diffusion. Standard deviations from the average are too small to visualize. Right: radial position-dependent water diffusion coefficients indicating a general increase in the diffusion of water with increasing distance from the particle center.

The incomplete phase separation of the organic material is likely to impact the bulk properties of the SSA models. To evaluate the impacts on mass transport properties, the mean squared displacement (MSD) and the corresponding diffusion coefficient (*D*) were calculated over the final 50 ns of each replicate for all of the water in the system. The results, given in Figure 5, show chemistry- and position-dependent variations in *D*.

Due to the uneven distribution of organics, *D* was evaluated for water molecules enclosed within unique volumes defined by spherical shells, similar to the geometric delineations given in Figure 4. In this way, we were able to correlate the location of long-lived organic clusters to the molecular diffusion of water. Generally, *D* is the lowest within 10 nm of the particle center and increases closer to the surface. One unique exception is system A2 (see Figure S4 for snapshots) in which the core region has a diffusion coefficient similar to the system average. This exception may be due in part to the fact that this replicate has fewer FAs situated in the core than other systems. Whereas A1 and A3 have a large FA agglomerate in the center, A2 has more FAs distributed near the surface. FAs may thus impede the diffusion of water by confining the water to smaller volumetric regions inside the particle, imposing physical membrane-like barriers throughout the aerosol. In larger SSA systems, FAs and other surfactants may take the form of vesicles and micelles, trapping internal water molecules and slowing diffusion.

The most complex systems (C) had the lowest apparent rates of diffusion. In C systems, the saccharides slow the diffusion of water through various intermolecular interactions. Highly soluble glucose monomers dispersed throughout the aqueous phase slow water diffusion via hydrogen bonding. Additionally, C3 contains laminarin which, as seen in SI Movie M5, interacts with neighboring LPS and BCL molecules to form a molecular aggregate. The aggregates slow water diffusion by creating physical barriers to movement and also by integrating into hydrogen bonding networks and increasing ion–water interactions, leading to the lowest *D* for all systems studied. LPS and divalent cations alone are known to form rigid gel-like structures through noncovalent ion bridging, preventing the diffusion of small molecules.^122^ However, our B systems do not show a decrease in diffusion due to LPS and divalent cations alone. Instead, only when coupled with additional mono- and oligosaccharides do we see a decrease in *D*. While the sugars in LPS are linear, laminarin is highly branched, which may allow for it to better interlink among the various organics and could help explain the difference between LPS alone (B systems) and with additional laminarin (C3). This shows that the type of organics, their organization, and their interactions in the aqueous phase can all modulate mass transport throughout SSAs.

Furthermore, divalent cations are expected to reduce the diffusion coefficient of water in solution relative to monovalent cations due to stronger ion–water interactions, which leads to codiffusion of the solvation shell of water molecules along with each cation. We would thus expect to see a corresponding decrease in *D* for systems B and C with the addition of Ca^2+^ and Mg^2+^ to the aqueous phase. However, this addition of cation complexity in our simulations did not always lead to a reduced *D*. We attribute this to the heterogeneous distribution of organics, whereby one diffusion coefficient is insufficient to describe the full complexity of molecular transport throughout aerosol particles.

### Interfacial Heterogeneity

**Figure 6:**
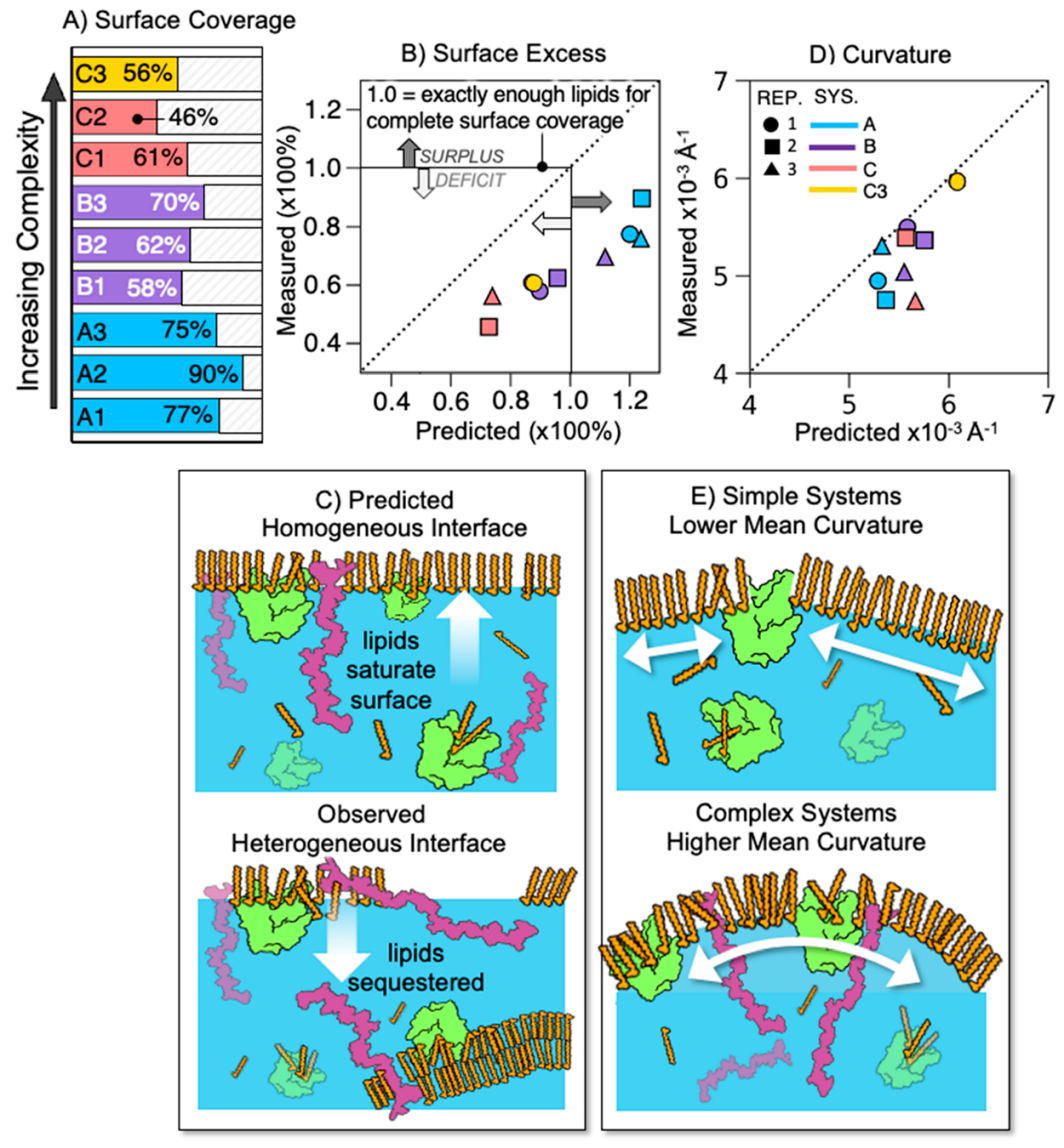
Surface coverage and morphology. (A) Surface coverage by fatty acids in order of increasing complexity. (B) Predicted vs measured surface excess. (C) Illustration of impacts of chemical complexity on surface morphology. (D) Predicted vs measured mean surface curvature of lipid rafts, weighted by the raft size or number of lipids per raft. (E) Illustration of the impacts of chemical complexity on surface curvature.

Finally, we wanted to understand how the distribution of the organic material impacts the morphology of the air/particle interface. We expected that the most hydrophobic components, the hydrocarbon FA tails, would completely saturate the surface in alignment with experimental observation and the core–shell theory. Using the clustering data extracted from the simulations, we identified the FA clusters that formed at the surfaces and estimated their total surface coverage (see the Supporting Information for calculation details). Figure 6A shows the percentage of surface area covered by FA monolayers compared to the total surface area of the final ellipsoid. As expected, systems with a more complex organic fraction had less FA surface coverage. The more complex systems have fewer total FAs, as they make up a smaller percentage of the overall organic mass fraction (A, 93%; B, 80%; and C, 63%).

For all systems, the percentage of the surface found to be saturated with FAs was lower than expected based on the total FA count. Because the percentage of fatty acids varies with each system, it is more pragmatic to evaluate the “surface excess” to better understand the extent to which chemical complexity impacts how the surface saturates with FAs. Here we define “surface excess” as the percentage of FAs covering the surface with respect to how many are available. We estimated how much of the surface should have theoretically been covered by lipids should all of the lipids available to the system have saturated the surface (Figure 6B). A value of 1 indicates that there are exactly the same number of lipids as there are available “sites” at the surface, where every surface site is occupied by a lipid. A value of >1 indicates that there are more lipids than surface sites available (surplus) and *vice versa* for values <1; i.e., a deficit means that there are fewer lipids than sites available for occupation at the surface. In all of the A systems, there are more lipids available than can fit at the surface, and we should expect 100% surface saturation. However, we observe that in each replicate there are available sites at the surface that are unoccupied by lipids. Even for the most complex systems (C), in which there is a lipid deficit for all replicates, fewer surfactants are present at the surface than would be expected.

This result can be attributed to the sequestering and stabilization of FAs in the core of the particle by other organics (Figure 6C). As a consequence, space at the surface is either left empty, exposing water directly to the atmosphere, or is occupied by other macromolecular species in the system such as BCL or LPS. While FAs spontaneously aggregate into aqueous structures, these structures are stabilized in solution by interacting with proteins and saccharides. BCL and LPS may be acting cosurfactants or emulsifying agents,^123−125^ enabling the system to maintain incomplete phase separation. Additionally, due to their amphiphilic nature, they also occupy space at the air/water interface, contributing to the observed lipid deficit and increasing interfacial heterogeneity.

An important observation from the analyses provided above is that the FAs accumulating at the surface do not do so uniformly; they aggregate into tessellated clusters with unique tilt angles, illustrated by the red and white patches visible in Figure 2. The clustered lipid patches, which are highly ordered, adopt a lower curvature than would otherwise be expected (Figure 6D,E) and are separated by rift-like “rivers” in which the lipids relax into a more disordered phase. This behavior can be attributed to the balance between the high bending modulus of the FA monolayer (i.e., energy required to perturb the equilibrium curvature) and the line tension of the curved particle surface. This phenomenon has been seen in previous laboratory studies of surfactant-coated microdroplets; surfactants accumulate into ordered domains at the interface and break apart into smaller, and presumably more planar, domains as the curvature of the interface increases.^126^

FA raft curvature increases with increasing chemical complexity, suggesting that the introduced macromolecules contribute to lowering the bending modulus of the monolayer. In the simple systems, the only perturbation to the rigid FA monolayers is BCL, which preferentially embeds between, rather than into, the FA rafts. In the more complex systems, the surfactant monolayers are perturbed by additional chemical species such as LPS, which increases interfacial heterogeneity and lends greater flexibility to the monolayers (Figure 6E). The lipid A region of LPS preferentially adsorbs to the surface at the rivers but is still able to insert into the ordered FA patches, likely due to the ability of FA-like alkyl chains on lipid A to align with the monolayer. This behavior has similarly been observed in previous studies of surfactants.^127−129^

**Figure 7:**
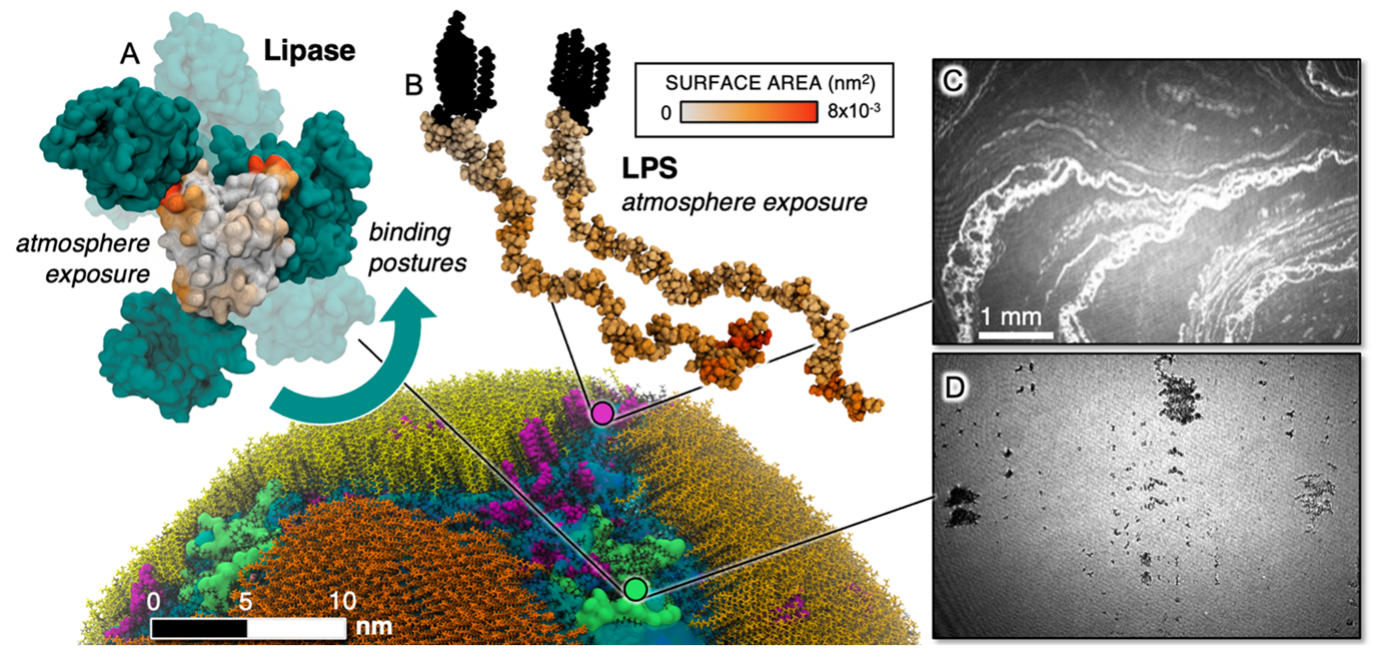
Fatty acid patches coat the particle surface, with other surface-active material disrupting the total surface coverage. Experimental BAM imaging supports model observations. Left: representative snapshot of C1 is shown (bottom) with FA clusters in yellow and orange, LPS in purple, and BCL in green. A representative BCL with other BCL enzymes (teal) bound to its surface is provided (A) to indicate the variety of BCL aggregates found across all simulations. The average residue exposure to the atmosphere is given on a white–orange–black scale for both (A) BCL and (B) LPS. Right: BAM imaging highlights the surface disruption of FA monolayers with either (C) LPS or (D) BCL at a surface pressure of ∼30 mN/m. These disruptions are seen as bright river-like regions of high FA content on the surface for (C), whereas (D) dark cluster regions, or holes, of low organic content are observed. In the absence of BCL and LPS, the FA monolayers show no such features at a surface pressure of 30 mN/m (see the Supporting Information for more details).

BCL and LPS are both observed at the aerosol interface and exhibit dramatic variations in interfacial morphology. To computationally evaluate the surface exposure of these two macromolecules, Figure 7A,B shows LPS and BCL colored by the frequency of atmospheric exposure, which highlights the most surface-active regions of each molecule. To corroborate our findings experimentally, we conducted Brewster angle microscopy (BAM) experiments in which we added BCL or LPS to monolayers of the FA mixture as described in the Methods section and in the Supporting Information. BAM images collected at a surface pressure of ∼30 mN/m are shown in Figure 7C. Bright regions of the images indicate aggregations of the FA molecules at the air/water interface, while dark regions indicate interfacial water as well as LPS molecules. In the absence of LPS and BCL, images are smooth at a surface pressure of 30 mN/m due to the high organic content (see Figure S6).

BCL (Figure 7D) appears to largely disrupt the fatty acid monolayers, as expected and observed previously.^72,73^ However, it also appears to aggregate, forming holes in the monolayer with little to no organized fatty acid density. We hypothesize that the tendency for the lipases to stick together to form clusters accounts for the holes (dark regions of low organic content) in the monolayer surface found in BAM images. To this end, we analyzed the frequency of finding BCL-BCL aggregates in our simulations and discovered a variety of aggregation postures. Figure 7A shows a selection of BCL-BCL conformations. It is possible that BCL aggregates together and sinks into the aqueous phase, having less affinity for the surface as a cluster. Interactions with FAs binding to exposed hydrophobic regions on the BCL surfaces may also contribute to lowered FA density in these regions, leading to the patchwork formations seen in Figure 7D.

LPS, on the other hand (Figure 7C), promotes river-like aggregates at the surface of high FA content surrounded by features containing LPS and water. The behavior seen here, although describing a phenomenon at a much larger scale than our simulations, is consistent with our computational observations. It is unclear, however, what the orientation of the LPS is at the interface. Our simulations suggest that while the lipid tail is much more surface-active, nearly the entire LPS molecule maintains some level of surface activity with regions of the sugar chain further from the lipid tail having greater atmospheric exposure than those closer (Figure 7B). The lipid A region can insert directly into and contribute to the bright regions with high FA density, while the saccharide headgroup can also adsorb to the surface and appear in the darker regions adjacent to the bright regions. Future studies are needed to help elucidate the mechanisms reported here.

Our work indicates that nascent SSAs are much more heterogeneous than traditional morphological models suggest, which may explain their high hygroscopicities. At the air/water interface, homogeneous long-chain saturated FA surfactants form a tightly packed monolayer that is known to have high rigidity, low fluidity, and impermeability to water.^130−134^ However, the insertion of additional molecular species such as LPS and BCL as in our experiments, or other biogenic marine cosurfactants such as alcohols, cholesterols, or shorter-chain FAs, results in a more porous, flexible, and fluid surface.^135−139^ Thus, despite having a significant mass percentage composed of organic material, the interface is not a thick organic coating as many models might suggest but rather a porous surfactant film that facilitates water uptake and evaporation.^140^ This explains how SSA particles are observed to have higher hygroscopicities than would be expected for the traditional viewpoint of particles fully coated by FAs.

Our simulations also provide insights into sustained enzymatic activity in SSA. BCL specifically has been observed in previous work to embed into lipid monolayers, with the consequence that it is directly exposed to the atmosphere.^84^ In addition to embedding at the surface, we show that BCL embeds into FA agglomerates in the core and aids in sequestering FAs away from the interface. BCL stability at surfactant monolayers is dictated by surfactant type and solution pH, with charged surfactants (such as DPPC, also present in the marine surfactant composition^140^) conferring more stability to the BCL-embedded monolayer than uncharged FAs.^83,84^ The enzyme is ultimately exposed to a variety of chemical microenvironments which are likely to impact its activity. Burris et al. demonstrated that BCL activity in microdroplets was increased 100-fold compared to the solution phase, hypothesizing that interfaces, mediated by particle coalescence, play a role in enzyme activity.^141^ Our simulations show that interfaces occur readily throughout each particle, suggesting an additional mechanism for the increased BCL activity associated with microdroplets.

### Extrapolating to Longer Time and Length Scales

Regarding the extrapolation of our findings to longer time and length scales, we believe that these results can be safely extrapolated to SSA diameters of up to 200 nm, which have the same or similar organic content. For submicrometer SSAs, specifically those <200 nm in diameter, the organic:inorganic mass ratio is >4:1, and for even smaller particles, this ratio can increase to ∼44:1.^16,78^ This mass ratio is primarily governed by the production mechanism of the particle. As the SSA diameter increases above 200 nm, the ratio changes dramatically toward higher inorganic content (reaching >80% inorganics) due to a shift in the dominant SSA production mechanism for larger particle sizes. For example, submicrometer SSAs, like those in our study, are traditionally associated with film drops and contain more water-insoluble material, while jet drops produce supermicrometer particles and tend to contain more inorganic species.^93^

The high organic content of submicrometer SSA produced via bursting film caps and the diversity in chemical properties of the organic content lead to the unique morphologies we see in this study. We expect that a moderate increase in diameter, of up to ∼200 nm, would lead to only subtle differences in the interfacial and bulk morphologies. This size increase would cause (1) a decrease in the particle curvature and (2) a decrease in the surface area-to-volume ratio. A decreased curvature caused by water uptake has been shown experimentally to facilitate the growth of surfactant lipid domains on microdroplets.^142^ The tessellated rafts seen in our simulations would likely increase in size but would remain as separate domains. Furthermore, BAM images indicate that we would still expect the same interfacial morphologies with respect to LPS and BCL as the surface curvature approaches zero.

Increasing particle size would also decrease the surface area-to-volume ratio, yet this would not result in significant morphological or rheological differences assuming all other factors remain constant. The FAs would still self-assemble into amorphous oil droplets and bisecting bilayer structures, which is characteristic for FAs at such low pH.^143−145^ FA vesicles are also formed at low pH in the aqueous phase; however, FAs tend to form vesicles with diameters ranging from 50 nm to 1.5 μm.^146,147^ With additional organics disrupting the highly ordered surfactants, it is possible that full vesicle formation could also occur in submicrometer SSA. Additionally, LPS is known to self-assemble into bilayer structures in the aqueous phase, but the thickness of one LPS bilayer approaches 200 nm; it is thus more likely that LPS will exhibit similar properties to those in the present study.

As time scales are extended, the overall morphology of these structures is expected to remain consistent. FA aggregation occurs rapidly (<50 ns) as demonstrated in SI Movie M1, with aggregates stabilized by strong hydrophobic interactions between tail groups. While we do not predict major structural differences to arise at extended times, small differences such as the reorganization of lipids within the aggregates themselves are likely to occur. This process is much slower, however, and unlikely to be observed via all-atom MD. For example, experimental and theoretical studies show that lipid raft formation in biological membranes occurs on the order of micro- to milliseconds.^148,149^

In addition, these simulations are representative models for nascent SSAs which are consequentially linked only to the early lifetime of these particles. Experimental work has shown that as nascent SSA age, their morphology drastically changes with exposure to gas-phase oxidants such as OH radicals and ozone.^150^ Because these particles exist in a dynamic environment where they constantly undergo compositional changes, a simulation of these particles for minutes to hours would not accurately represent the real lifecycle of nascent SSA. Thus, the simulations presented in this study represent the important metastable states that nascent SSAs develop during their early lifetime.

## Conclusions

We use ultralarge all-atom molecular dynamics simulations coupled with Brewster angle microscopy of submicrometer marine aerosols to understand the link between chemical complexity and aerosol morphology. We show that fatty acid surfactants readily aggregate and distribute to the surface and into oily aggregates within the aerosol. We find that rigid lipid monolayers at curved aerosol surfaces form discontinuous patches, separated by disordered regions in which amphiphilic species such as BCL and LPS can aggregate. The overall distribution of organic material throughout the particle is consistent across variations in the organic-phase complexity, and in more complex particles, less of the aerosol surface is covered. We argue that organic SSAs may not always phase separate to adopt a core–shell morphology where the aerosol surface is coated with a thick organic layer. Rather, we propose that organic SSA particles containing the full chemical complexity of biogenic marine molecules adopt more heterogeneous morphologies, enabling them to readily take up water despite being largely organic. How this study informs the complexity of other aerosols including those produced from the human respiratory tract remains to be uncovered.

Our work informs upon and paves the way for future studies of aerosols with computational microscopy that are not just limited to sea spray. The COVID-19 pandemic highlighted the importance of aerosols in the transmission of airborne pathogens between individuals.^151−153^ It has been observed that submicrometer rather than supermicrometer aerosols are more likely carriers of active SARS-CoV-2.^153^ Insights from our work suggest that morphological heterogeneity may play a role in stabilizing regions of physiological relevance within a respiratory organic aerosol, aiding in the protection of viruses during airborne transport. Future studies of aerosols in this size regime will be useful in illuminating the mechanisms behind the airborne transmission of enzymes, viruses, and bacteria throughout the environment.

## Supporting Information Available

- Movie M1: system A1 surface view, initial 50 ns (MP4).
- Movie M2: system A1 surface view, final 50 ns (MP4).
- Movie M3: system B1 cross section, final 50 ns (MP4).
- Movie M4: system B1 surface view, final 50 ns (MP4).
- Movie M5: system C3 cross section, final 50 ns (MP4).
- Supplemental figures, tables, calculations, full SI movie descriptions, additional experimental (BAM), and computational design details (PDF)
- Transparent Peer Review report available (PDF)
