# Supplementary material for: Revealing the Impacts of Chemical Complexity on Submicrometer Sea Spray Aerosol Morphology
Source: ACS Cent Sci. 2023 May 4;9(6):1088–103. doi: 10.1021/acscentsci.3c00184 (PMC10311664; doi:10.1021/acscentsci.3c00184)
Supplement: Supplementary file 6 — oc3c00184_si_007.pdf [file oc3c00184_si_007.pdf]

Supporting Information for: Revealing the Impacts of Chemical Complexity on Submicron Sea Spray Aerosol Morphology

Abigail C. Dommer,[1] Nicholas A. Wauer,[1] Kyle J. Angle,[1] Aakash Davasam,[1] Patiemma Rubio,[1] Man Luo,[1] Clare K. Morris,[1] Kimberly A. Prather,[1] Vicki H. Grassian,[1] Rommie E. Amaro[1*]

[1]Department of Chemistry and Biochemistry, University of California, San Diego, La Jolla, CA 92093, USA

*Corresponding Author: Rommie Amaro, Email: ramaro@ucsd.edu

Table of Contents

Figures

Figure S1: Sample SSA Morphologies S2
Figure S2: Laminarin Molecular Structure S2
Figure S3: NAMD Efficiency Scaling S3
Figure S4: MD Snapshots of All Systems S7
Figure S8: Ellipsoid Diagram S11
Figure S9: Example MSD Plot S12
Figure S10: Sample Sphere Fits S13
Figure S11: Spherical Cap Diagram S14

Tables

Table S1: SSA Ion Concentrations S4
Table S2: Atom Count and Simulation Lengths S6
Table S3: O:C and H:C Ratios by Simulation S6
Table S4: Equations for Error Propagation S12

Computational Experimental Design

Ion Selection S3
Organics Selection S4
Water Content S6
Systems and Timescales S6

Brewster Angle Microscopy Experimental Details

Description of BAM S7
Figure S5: Schematic of BAM experimental set-up S8
Figure S6: BAM image series for Mix-FA monolayer compression S9
Figure S7: Isotherms for Mix-FA monolayer with LPS S9

Calculations

Asphericity and Relative Shape Anisotropy S10
Organic Distribution by Volume S10
Mean Square Deviation and Diffusion S11
Surface Curvature S12
Surface Area Coverage S13
Ellipsoidal Surface Area S14
Error Propagation S14

Movies

Movie M1: System A1 Surface View, Initial 50 ns S15
Movie M2: System A1 Surface View, Final 50ns S15
Movie M3: System B1 Cross Section, Final 50 ns S15
Movie M4: System B1 Surface View, Final 50 ns S16
Movie M5: System C3 Cross Section, Final 50 ns S16

Figures

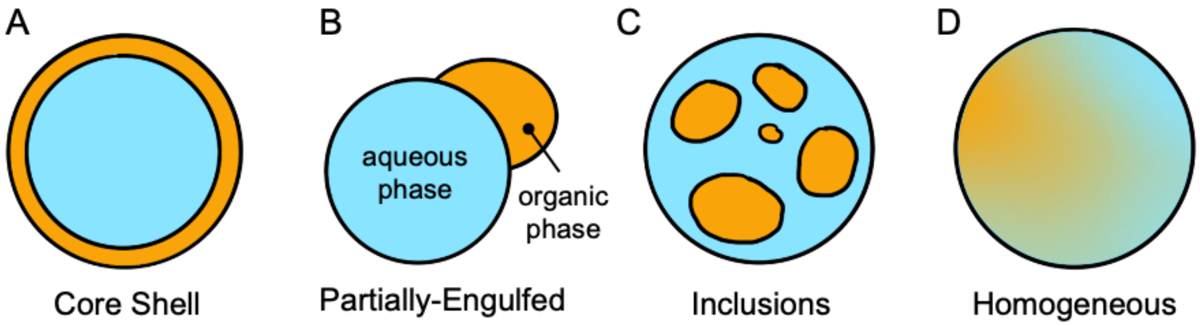

Figure S1. Different morphologies observed for phase separated or homogeneous mixed organic/aqueous particles. Orange and blue colors represent organics and water, respectively.

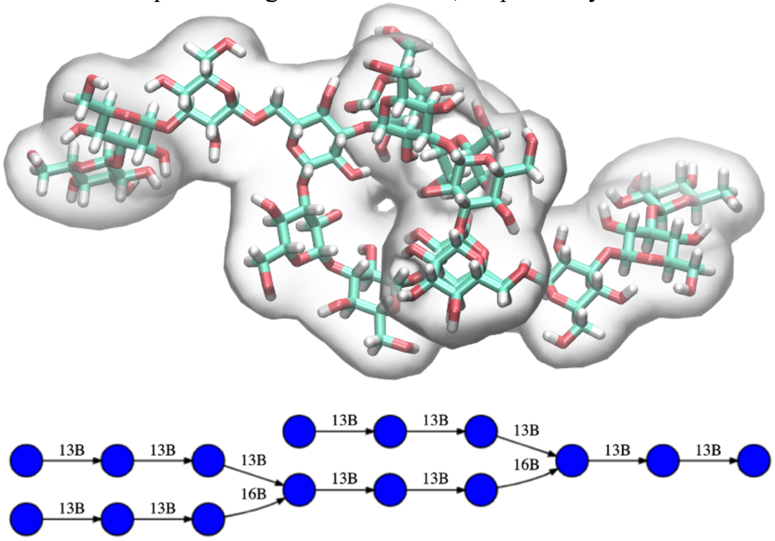

Figure S2. Molecular structure of glucose oligosaccharide, laminarin. Since laminarin has a variety of molecular weights and branching ratios, this complexity is impossible to recreate computationally. Only structure was generated to represent neutral, branched oligosaccharides. See **Main Text** for details.

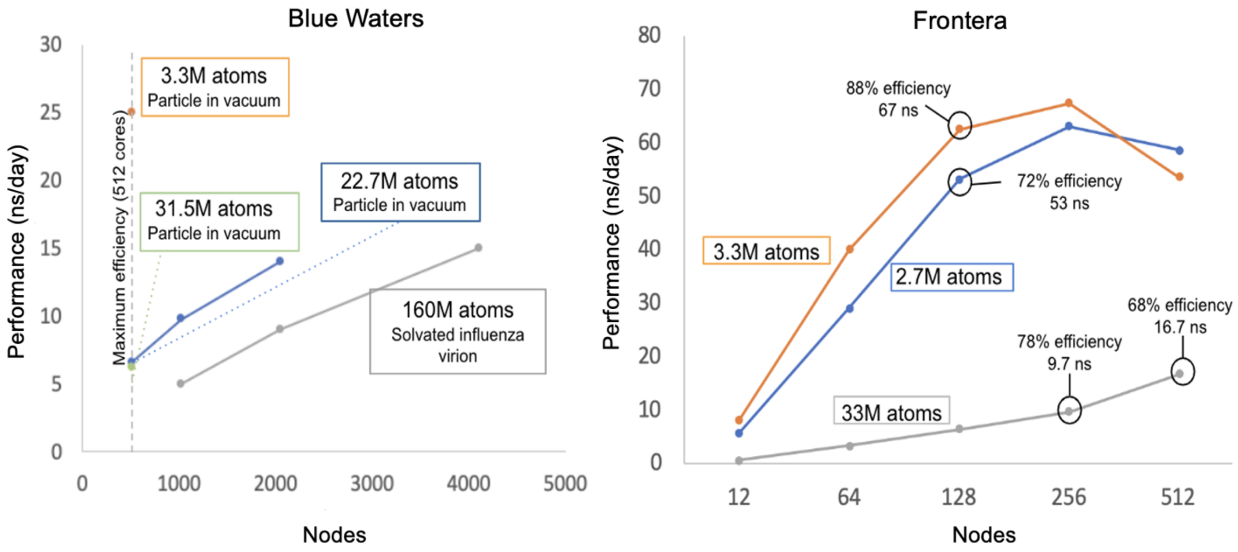

Figure S3. NAMD efficiency scaling across UIUC Blue Waters and TACC Frontera. Note scale differences.

Experimental Design

The chemical components of the SSA models were selected based on the most up-to-date molecular analyses of nascent submicron SSA; although, it is worth noting that at the time of writing, only an estimated 25% of the total chemical species found in SSA has been fully characterized.[1] We aimed to reasonably represent in our models 1) the chemical composition of ions; 2) the chemical composition of organic matter; and 3) the mass percentage of water, which is variable based on hygroscopic growth factor and relative humidity.

1) Chemical composition of ions.

Elemental analysis of water-soluble ions by ion-exchange chromatography has identified the major ionic species in small particles with dry diameter d_p = 150 nm,[1,2] given in **Table S2**. We considered the accuracy of the CHARMM36 force field when selecting which ions to incorporate into our models. Sodium is commonly used in MD simulations given its biological relevance, and its interactions with TIP3P water, protein, lipids, and chloride ions have been experimentally validated below concentrations of 1 M.[3] The concentration of sodium in bulk seawater and SSA is approximately 0.46 M, falling within the accuracy range.

Table S1. List of most common ionic species found in fine SSA (150 nm) ordered by decreasing mass percentage of a wet particle at 80% relative humidity. For more information, see Bertram et al.[1]

Ionic species	Mass percentage
Cl^-	4.9
Na^+	2.7
SO_4^{2-}	0.81
Mg^{2+}	0.38
Ca^{2+}	0.14
K^+	0.11
Br^-	0.035
**Total**	9

The interactions between Na^+, Ca^{2+} and Mg^{2+} with carboxylate, phosphate, and sulfate groups are more complicated, but were of greatest interest to our study. Force fields for non-polarizable divalent metal cation models have been notoriously inaccurate. Recent work has addressed this challenge to improve Mg^{2+} and Ca^{2+} interactions with a variety of macromolecules, including the TIP3P water model, with reasonable experimental consistency.[4–7] Specifically, experimentally-validated fixes for Na^+ and Ca^{2+} interactions with carboxylates (of interest to our systems) have been added to the default CHARMM36 force fields, are now widely used, and reproduce experimentally-observed behavior.[6,8–10] While adjustments to the default parameters for Mg^{2+} are under discussion, we elected to include Mg^{2+} without any additional modifications for simplicity and easy comparison to the existing computational literature.[7,8,11] Carbohydrate-linked phosphates and sulfates have also been validated and incorporated into the CHARMM36 force field parameter set used in this work.[12] Sulfate and bromide anions were not included in our models due to a relative lack of force field validation in the literature.

2) Chemical composition of organic matter.

There is a general consensus on the major classes of organic molecules observed in SSA, although their relative abundances are known to vary with ocean productivity, temperature, and geographic region.[1,2,13–16] Molecular and elemental characterization in Cochran et al. identified major submicron SSA types to contain 1) siliceous material (e.g., diatomaceous fragments); 2) long-chain fatty acids; 3) short-chain fatty acids; 4) free saccharides and polysaccharides; and 5) fluorescent humic-like substances.[13] Kirpes et al. reported a category

for amino acids, which is consistent with other reports of free and combined amino acid enrichment at the sea surface microlayer and within SSA.[14,17–19] Additional categories of organic matter could be argued to include alcohols, sterols, phospholipids, and hydrocarbons, but are omitted from this work for simplicity.[1,20,21]

Thus, the most abundant and consistently-reported components across ambient marine aerosol field observations are fatty acids, protein, saccharides, and humic-like substances. Humic acids are water soluble, macromolecular fluorescent compounds of biological origin, with molecular weights, connectivities, and compositions, all of which vary with biological processing. Since a specific molecular structure for humic acid was unavailable at the time of writing, and due to the complexity of its analysis and parameterization, it was not included in our models. The remaining major categories of organics are, however, represented in this work. Fatty acid chain lengths, including long and short chains, as well as their relative ratios, were selected based on anionic surfactant speciation by Cochran et al.[21] The protein we incorporated is *Burkholderia cepacia* (BCL) lipase, which has been found to retain its activity in nascent SSA[22] and has been extensively modeled and characterized by our group and others.[23–27] We then incorporated three classes of saccharides: anionic polysaccharides (lipopolysaccharides), neutral oligosaccharides (laminarin), and free monosaccharides (glucose), each of which has specific and unique properties that can influence SSA phase, shape, viscosity, and reactivity.

We note that all fatty acids here are fully protonated to reflect the low pH environment (<6)[28] of SSA and the air/water interface.[29,30] The fatty acid protonation state and the lack of additional surfactant diversity (e.g., unsaturated fatty acids, cholesterol, and phospholipids) give the monolayers and aggregates unique properties that may not necessarily reflect the true properties of the lipids in natural SSA. For example, fully protonated fatty acids are largely insoluble, and their monolayers are rigid and gel-like, especially without biologically-derived double bonds which would increase monolayer fluidity.[31] Additionally, protonated fatty acids preferentially aggregate into oil droplets in the aqueous phase, whereas anionic forms tend to assemble into micellar and lamellar structures.[31,32] The pH-dependent properties of FAs may have interesting consequences for aerosol morphology; as aerosols acidify over time, FAs may undergo dynamic rearrangement into more planar, homogenous, monolayers with higher viscosity and lower porosity. Additional studies on the impact of protonation state on FA mixtures within submicron SSA are underway.

3) Water content.

The water content of SSA depends on a variety of factors, including size, age, hygroscopic growth factor and relative humidity. The water content of the SSA in this work was based on the estimates derived by Bertram et al. for freshly-emitted SSA at a relative humidity of 80%[1].

4) Timescales.

We are interested in investigating the impact of chemical complexity on SSA morphology and dynamics, specifically for those systems sizes which are too small to reliably experimentally validate. We selected a timescale of 500 ns – 1μs for our simulations based on small-scale studies of fatty acid aggregation and mixing. The time-resolved information gathered from particle shape evolution and clustering analyses (**Figures 2** and **3**) indicate that these parameters reach consistent values after ~200 ns.

Table S2: Atom counts and total simulation time for each system.

	A1	A2	A3	B1	B2	B3	C1	C2	C3
Atom count ($\times 10^6$)	3.083	2.907	2.933	2.649	2.462	2.731	2.102	2.670	2.546
Simulation time (ns)	1,000	515	502	533	574	530	1,000	597	593

Table S3. Elemental number ratios of each system.

	A1	A2	A3	B1	B2	B3	C1	C2	C3
O:C	0.133	0.133	0.133	0.192	0.192	0.192	0.299	0.299	0.262
H:C	1.98	1.98	1.98	1.95	1.95	1.95	1.94	1.94	1.93

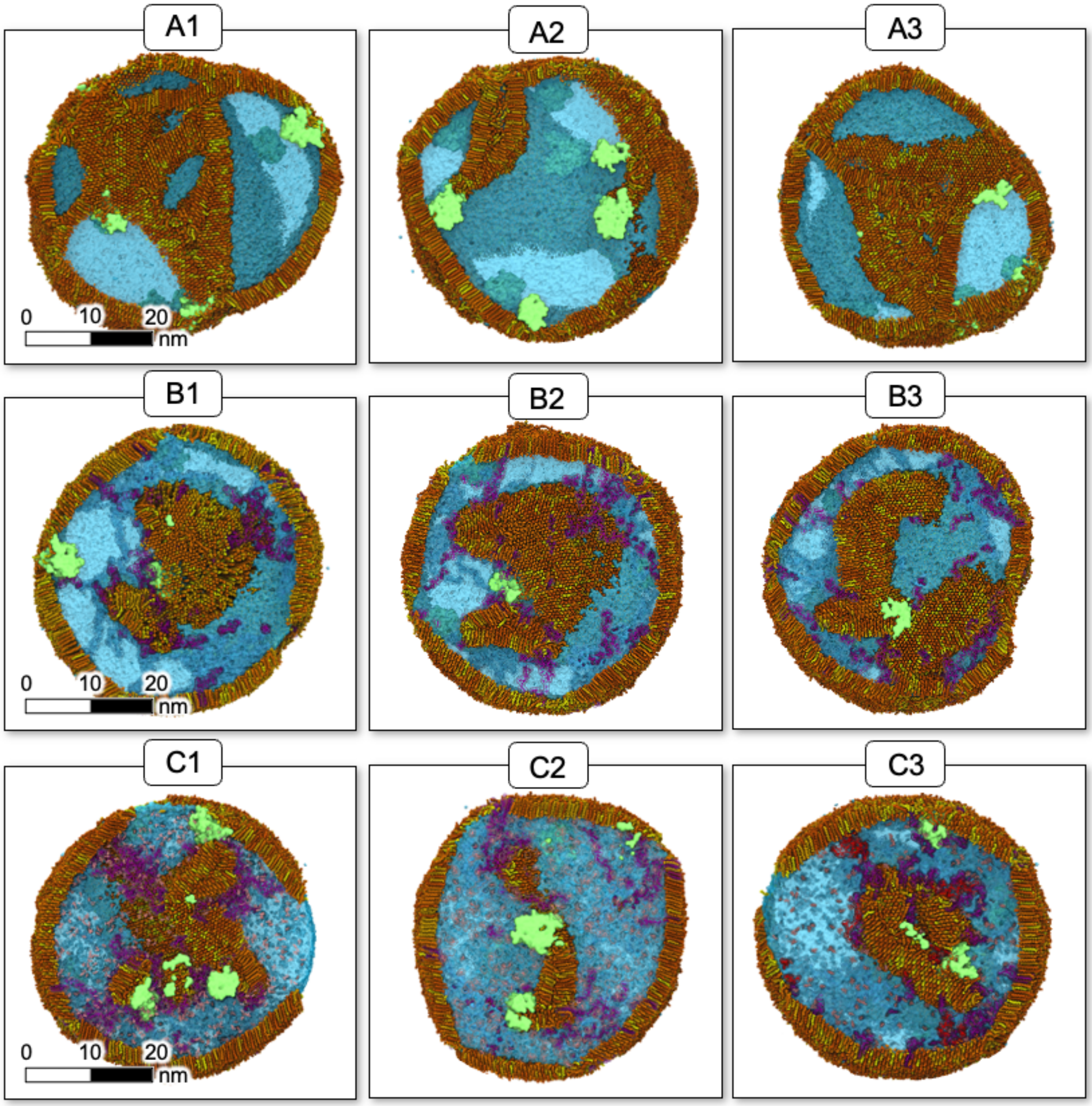

Figure S4. Cross-Sections of each replicate, final frame, visualized by VMD. LA, MA, PA, and SA are given in orange and yellow VdW representation, BCL is given in QuickSurf lime, LPS is given in purple licorice; glucose in pink licorice, and laminarin in red licorice. Water is represented in transparent dark cyan QuickSurf.

Brewster Angle Microscopy Experimental Details

The following discussion and **Figures S5-7** are reproduced from the dissertation of Man Luo.[33] Brewster Angle Microscopy (BAM) is a non-invasive optical technique for surface characterization on a large, millimeter dimensions. It has been widely used for characterizing one molecule thick monolayers at the air/water interface. The BAM uses p-polarized light at the Brewster Angle of incidence. At the Brewster angle, at the air/water

interface there is no reflection from the air/water interface and the background is dark. When a condensed phase of a monolayer with a different refractive index is present at the air/water interface, light reflection will occur, and a high contrast image can be generated. Overall, BAM can be used to investigate the morphology of the monolayer at the air/water interface by examining dark and light regions of the surface.

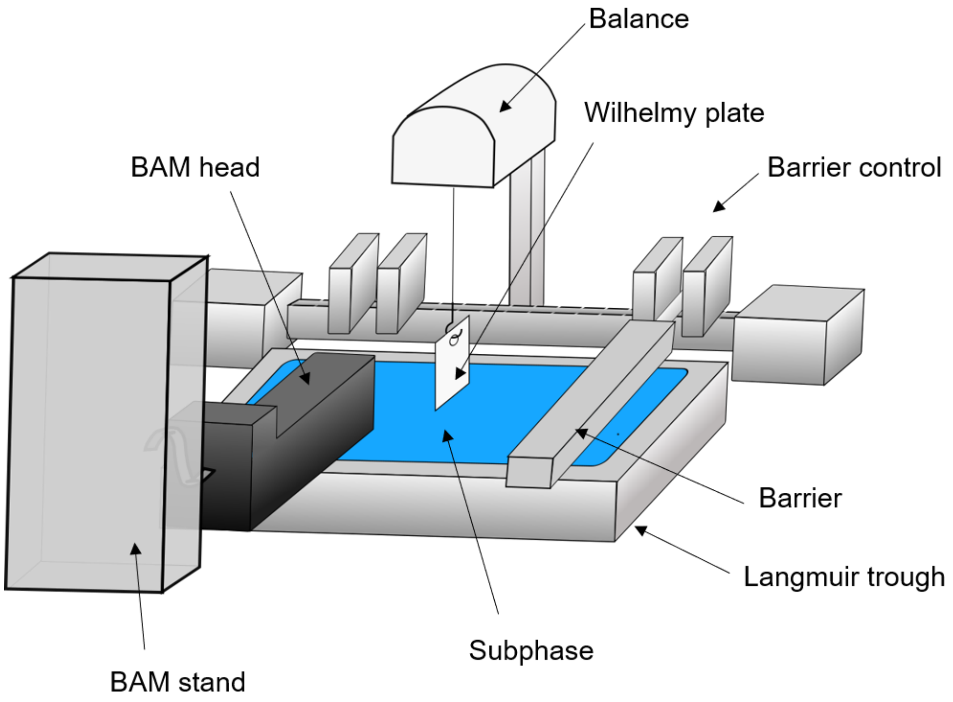

Figure S5: Schematic of the BAM coupled with the Langmuir trough.

A schematic of the BAM setup with the Langmuir trough is shown in **Figure S5**. Using this setup, BAM images can be obtained during the monolayer compression process. Therefore, the morphology change of the monolayers during compression can be studied. One barrier was taken off from the trough in order to place the BAM instrument and the remaining barrier was used to compress the monolayer.

BAM images obtained during compression provide insights into surface monolayers. **Figure S6** shows an example of how mixed FA monolayers (here termed "Mix-FA" and composed of the 1: 2: 4: 3 ratio described in the Experimental Methods section) can be disrupted by LPS in the subphase. In this experiment, BAM images are collected as the monolayer is compressed up until a surface pressure of 30 mN/m. These images are shown at surface pressures before the complete collapse of the monolayer (see **Figure S7**).

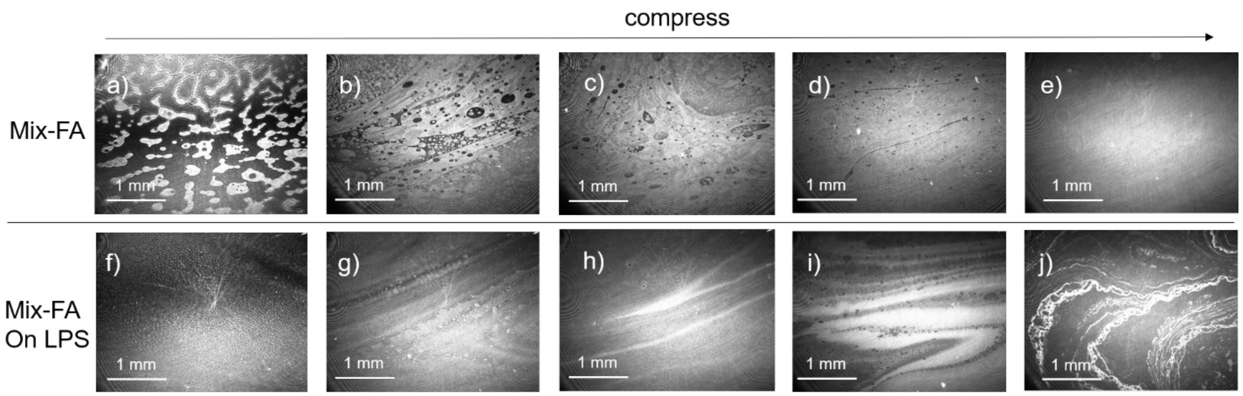

Figure S6: Example of BAM images for Mix-FA monolayer on 0.4 M NaCl subphase (a-e) during compression in the Langmuir from 0 mN/m to 30 mN/m (0, 1, 5, 15 and 30 mN/m images shown). At 30 mN/m (e), the surface looks “smooth” without rivers or rafts. The same experiment done on a NaCl subphase containing LPS (f-j). At 30 mN/m, river-like regions of high (bright regions) and low (darker regions) organic content are present at the surface as discussed in the main text.

The isotherm for the above systems can be seen from **Figure S7**. It can be observed that the isotherm of the Mix-FA monolayer is similar to a typical fatty acid isotherm and shows the collapse of the monolayer above 30 mN/m. However, with the presence of LPS in the subphase, the lift-off point (where surface pressure starts to rise above 0 mN/m) is significantly shifted toward higher mean molecular area, indicating the insertion of LPS molecules in the monolayer at the air/water interface. There is no distinct phase but a gradually increased surface pressure during the compression of the Mix-FA monolayer on the LPS subphase, which indicates that the LPS molecules at the air/water interface insert into and disrupt the Mix-FA monolayer. This is a key finding that demonstrates the computational observation of LPS at the interface can be measured (at a different scale) experimentally.

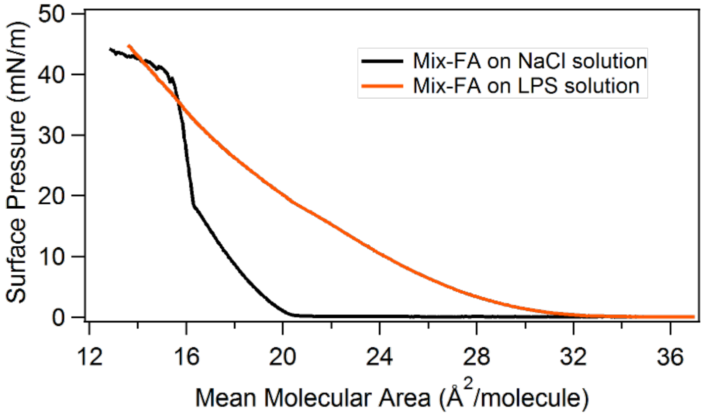

Figure S7: Isotherms for Mix-FA monolayer on 0.4 M NaCl subphase and when LPS is added to the salt solution subphase.

Calculations

1) Asphericity and Relative Shape Anisotropy.

Here, we use the definition of asphericity derived by Theodorou and Suter:[34]

$$\phi = X^2 - \frac{1}{2}(Y^2 + Z^2),\ \phi \geq 0. \quad \text{(SE1)}$$

The quantities X^2, Y^2, and Z^2, are given by the symmetric radius of gyration tensor,

$$S = \begin{bmatrix} X^2 & XY & XZ \\ XY & Y^2 & YZ \\ XZ & YZ & Z^2 \end{bmatrix}, \quad \text{(SE2)}$$

such that the eigenvalues are $X^2 \geq Y^2 \geq Z^2$. S, derived from the definition

$$S_{m,n} = \frac{1}{N}\sum_{k=1}^{N} r_m^k r_n^k \quad \text{(SE3)}$$

where N represents the number of atoms in the system, $m \in \{1,2,3\}$, and $n \in \{1,2,3\}$, is computed directly from atomic coordinates. The squared radius of gyration, R_0^2, is given by the first invariant of S,

$$R_0^2 = X^2 + Y^2 + Z^2, \quad \text{(SE4)}$$

and is used to calculate the relative shape anisotropy, κ^2. This term is a shape descriptor of the symmetry of the particle; that is, a value of 1 indicates a rod-like symmetry, where all atoms lie along a line, while a value of 0 indicates the particle has a higher degree of symmetry, such as that of a perfect tetrahedron or sphere.[1,3] Here, we use the definition of κ^2 given by Theodorou and Suter:[1]

$$\kappa^2 = \frac{\phi^2 + \frac{3}{4}(Y^2 - Z^2)^2}{R_0^4}. \quad \text{(SE5)}$$

A close inspection of this equation reveals that the quantity in the numerator is a function of the asphericity and the parameter *acylindricity*, given by $Y^2 - Z^2$, where a value of zero indicates perfect cylindrical symmetry. A more detailed description of these quantities is out of the scope of this work but can be found in the provided references.

2) Organic Distribution by Volume.

To quantify distribution of organic material throughout the particle, we calculate the best-fit ellipsoid to the dataset. Let *n*, M be the total particle mass, and *a, b,* and *c* correspond to the dimensions of each semi-axis, as illustrated by **Figure S8**. *I* can be calculated using the internal *measure inertia* command in VMD,[35] which returns the eigenvalues and the principal axes.

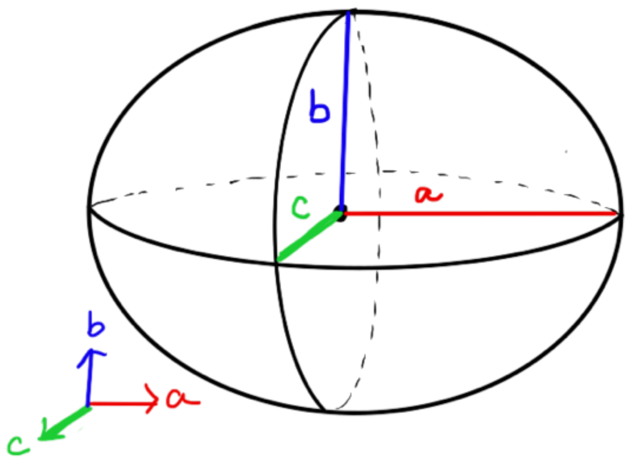

Figure S8: Sketch of an ellipsoid with axes of symmetry labeled.

We then subdivide the ellipsoid into three regions by volume, termed "core," "bulk," and "shell," using the approach outlined by Karadima et. al.[36] Drawing concentric ellipsoids along the principle axis each enveloping approximately a third of the volume, we can describe the distribution of mass by type into each region of the particle.

3) Mean Square Deviation and Diffusion.

Here, we computed the diffusion coefficients for water across the various systems using the Einstein formula to calculate the mean squared displacement (MSD),

$$MSD(r_d) = \langle \frac{1}{N} \sum_{i=1}^{N} |r_d - r_d(t_0)|^2 \rangle \quad \text{(SE6)}$$

where N is the number of equivalent particles, r is their coordinates, and d = 3 for movement in 3 dimensions. This, specifically, was done utilizing MDAnalysis's tool EinsteinMSD.[37–39] The diffusion coefficient, D, is related to the MSD by equation (SE7). Performing linear regression on the "middle" region of the MSD curve allows for the estimation of the diffusion coefficient from the slope of the linear regression. The "middle" region can be described as the linear region of a log plot of the MSD.[40] For this work, it was defined as from 1-9 ns of

the MSD calculation as the MSD above 10 ns can deviate from linearity **(Figure S9).** The TIP3P water model tends to over-estimate the rate of diffusion due to the lack of longer-range water structure. Despite this our calculations still yield diffusion coefficients significantly lower than both computationally determined TIP3P values and the experimentally determined values of pure water at 298 K.[41] The spherical shell radii were selected semi-arbitrarily to give similar numbers of water molecules in each shell and to account for the asphericity observed in certain systems. Each of the selections contains at least 40,000 water molecules used for the MSD calculations.

$$D_d = \frac{1}{2d} \lim_{t \to \infty} \frac{d}{dt} MSD_{(r_d)} \quad \text{(SE7).}$$

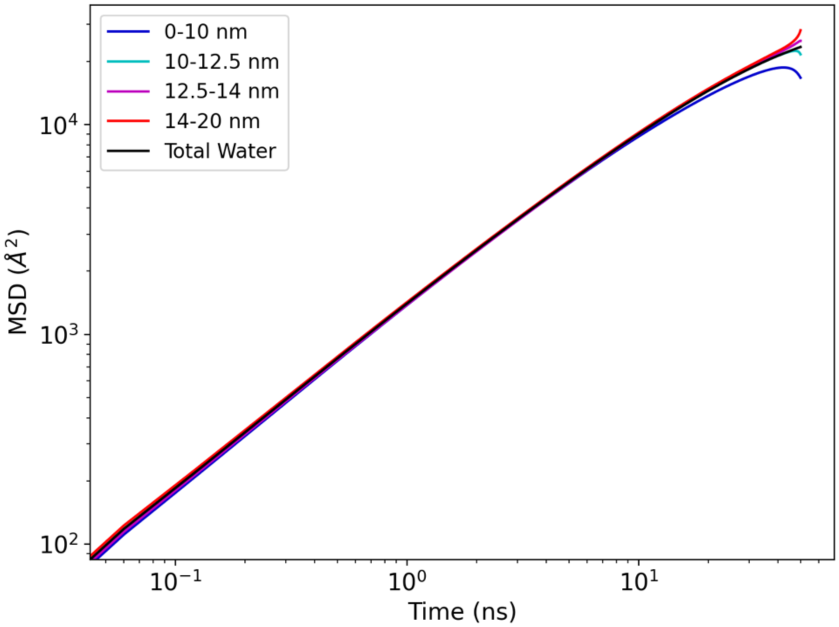

Figure S9: Example log plot of MSD from C3. The core region deviates from linearity after 10 ns leading to the 1-9 ns region being defined as the "middle" region for diffusion coefficient calculations.

4) Surface Curvature Estimation.

Here, we use the inverse radius of the best fit sphere as our definition of curvature. That is, for the given set of datapoints, we find the best fit sphere using a NumPy linear algebra least squares function. In this case, the data points are the points defined by the fatty acid headgroups. **Figure S10** shows some example sphere fits to lipid selected lipid clusters.

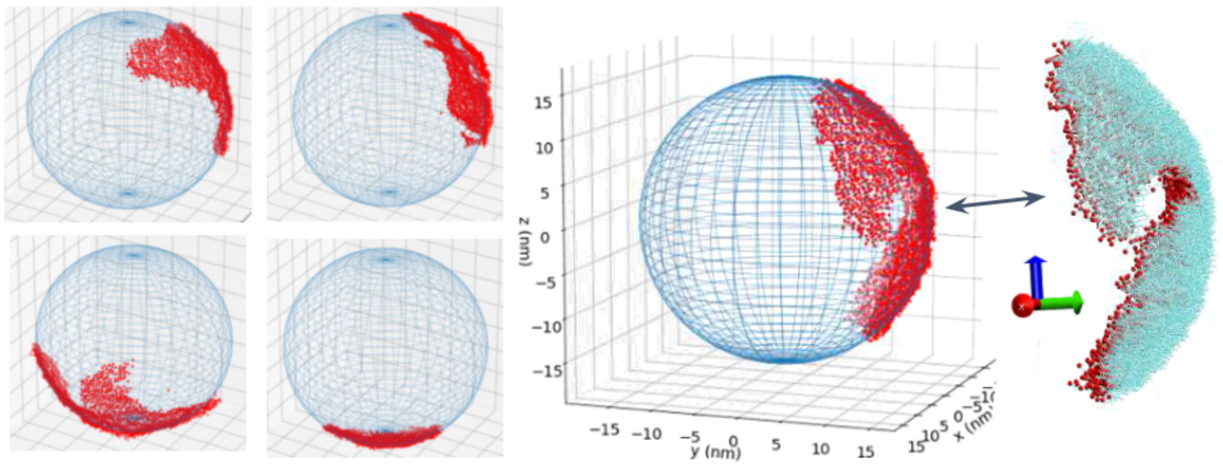

Figure S10: Spherical fits to selected lipid clusters to demonstrate the sphere fit function.

5) Estimation of Surface Area Coverage.

To estimate the total surface area, we must first know the area per lipid of the particular surface cluster. Surface clusters were identified manually by visual inspection. The estimation of area per lipid is non-trivial. There are many methods one could use for this calculation, but the procedure we followed is enumerated below.

1. The radial distribution function (RDF) was calculated between the headgroups (the first carbon atoms) using MDTraj.[42] This allows us to extract the average distance between headgroups.

2. Using the RDF plot, we used NumPy[43] to fit a gaussian curve of the function

$$g(x) = \frac{1}{\sigma\sqrt{2\pi}} \exp\left(\frac{-(x-\mu)^2}{2\sigma^2}\right) \qquad \text{(SE6)}$$

to the first peak, which corresponds to the distribution of nearest headgroups. We then extract the mean value (μ) as the radius and the standard deviation (σ) as the error associated with the calculation.

3. Using the radius r of the best fit sphere to the lipid cluster calculated above, we can estimate the average area covered by a lipid on a curved surface using the geometric principles of a spherical cap, illustrated in **Figure S11**, where the distance between two lipid headgroups is defined as $2a$.

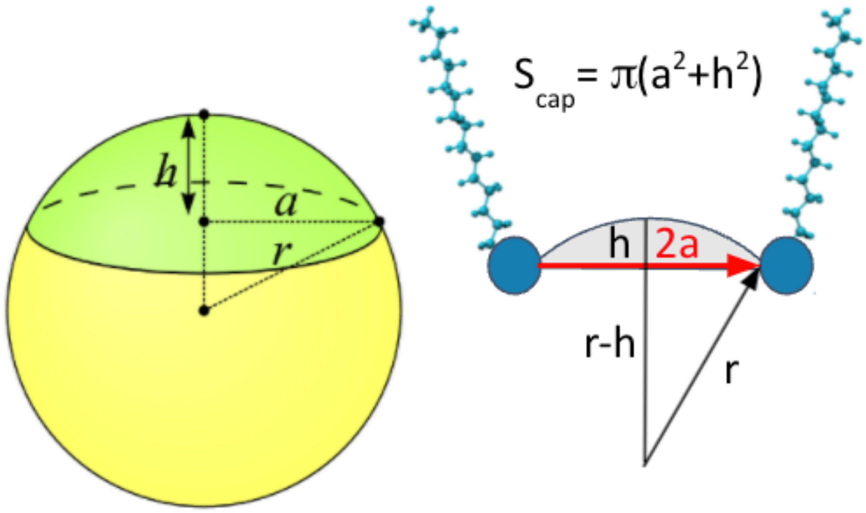

Figure S11: Diagram of spherical cap calculation.

4. Multiplying the resulting area by the number of lipids in the cluster and propagating error appropriately gives the estimate of the total area covered by the lipid cluster.

6) Estimation of Ellipsoidal Surface Area Coverage.

One equation estimating the surface area of an ellipsoid is given by Knud Thomsen's Formula:

$$S \approx 4\pi \left(\frac{(ab)^p+(ac)^p+(bc)^p}{3}\right)^{1/p} \quad \text{(SE7)}$$

where the constant $p = 1.6075$ gives an approximate error $\leq 1.061\%$.[44–46] The estimated surface area can thus be calculated using the semi-axis values extracted from the best fit bounding ellipsoid calculation above.

7) Error Propagation

Error was propagated using the equations in **Table S4** for the analytical propagation of uncertainty.[47]

Table S4: Equations for the propagation of uncertainty.

Type of Analysis	Example	Equation	Eq
Addition/Subtraction	$x = a + b - c$	$s_x = \sqrt{s_a^2 + s_b^2 + s_c^2}$	SE8
Multiplication/Division	$x = a \times b \div c$	$\frac{s_x}{x} = \sqrt{(\frac{s_a}{a})^2 + (\frac{s_b}{b})^2 + (\frac{s_c}{c})^2}$	SE9

Movies

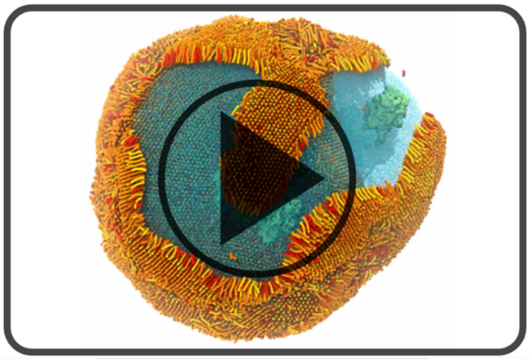

Movie M1: Surface view of System A1 during first 50 ns of simulation. Initial frames show randomly distributed components evolving during minimization, heating, and equilibration, followed by 50 ns of production. Orange, red, and yellow molecules represent fatty acids lauric, myristic, palmitic and stearic acids; water is given by blue dots, and lipase is given in lime green QuickSurf representation (VMD).

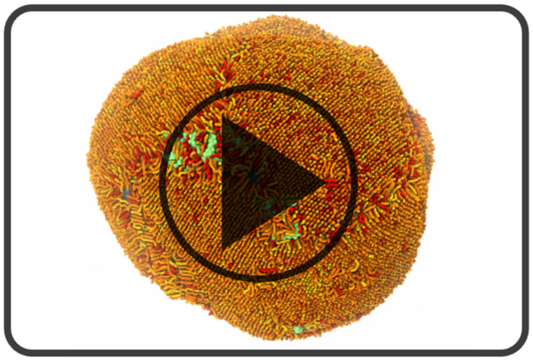

Movie M2: Surface view of System A1 during final 50 ns of simulation, showing nearly complete surface saturation by fatty acids (red, orange & yellow), with lipase (lime green) inserting in between fatty acid patches. Orange, red, and yellow molecules represent fatty acids lauric, myristic, palmitic and stearic acids; water is given by blue dots, and lipase is given in lime green QuickSurf representation (VMD).

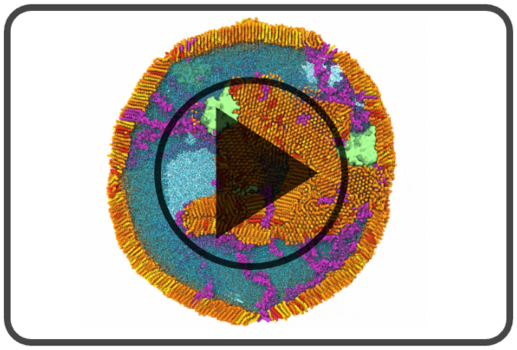

Movie M3: Cross section view of System B1 during final 50 ns of simulation, showing distribution of organic material throughout the center of the particle. Notable are fatty acids (red, orange & yellow) forming into bilayer and aggregate clusters inside the particle. Lipase (lime green) and LPS (purple) inserts both into fatty acid aggregates in the center and the monolayers at the surface. Orange, red, and yellow molecules represent fatty

acids lauric, myristic, palmitic and stearic acids; water is given by blue dots, lipase is given in lime green QuickSurf representation (VMD), and LPS is given in purple by VdW representation (VMD).

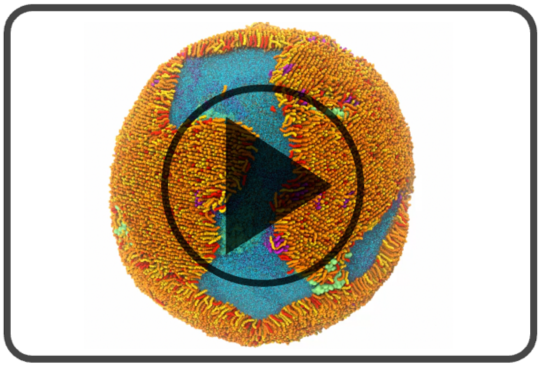

Movie M4: Surface view of System B1 during final 50 ns of simulation, showing incomplete complete surface saturation by fatty acids (red, orange & yellow), with lipase (lime green) and LPS (purple) inserting in between fatty acid patches. Orange, red, and yellow molecules represent fatty acids lauric, myristic, palmitic and stearic acids; water is given by blue dots, lipase is given in lime green QuickSurf representation (VMD), and LPS is given in purple by VdW representation (VMD).

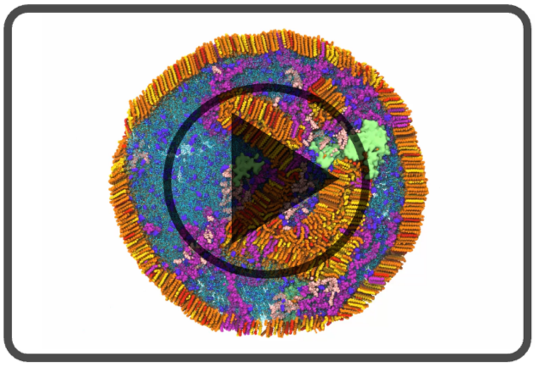

Movie M5: Cross section view of System C3 during final 50 ns of simulation, showing impeded molecular diffusion throughout the particle by LPS (magenta), protein (lime), laminarin (pink) and fatty acid (red, orange, & yellow) aggregation. Orange, red, and yellow molecules represent fatty acids lauric, myristic, palmitic and stearic acids; water is given by blue dots, BCL lipase is given in lime green QuickSurf representation (VMD), and LPS, laminarin, and glucose are given in magenta, pink, and indigo, respectively, by VdW representation (VMD).

References

(1) Bertram, T. H.; Cochran, R. E.; Grassian, V. H.; Stone, E. A. Sea Spray Aerosol Chemical Composition: Elemental and Molecular Mimics for Laboratory Studies of Heterogeneous and Multiphase Reactions. *Chemical Society Reviews* **2018**, *47* (7), 2374–2400. https://doi.org/10.1039/C7CS00008A.

(2) Jayarathne, T.; Sultana, C. M.; Lee, C.; Malfatti, F.; Cox, J. L.; Pendergraft, M. A.; Moore, K. A.; Azam, F.; Tivanski, A. V.; Cappa, C. D.; Bertram, T. H.; Grassian, V. H.; Prather, K. A.; Stone, E. A. Enrichment of Saccharides and Divalent Cations in Sea Spray Aerosol during Two Phytoplankton Blooms. *Environmental Science and Technology* **2016**. https://doi.org/10.1021/acs.est.6b02988.

(3) Venable, R. M.; Luo, Y.; Gawrisch, K.; Roux, B.; Pastor, R. W. Simulations of Anionic Lipid Membranes: Development of Interaction-Specific Ion Parameters and Validation Using NMR Data. *The Journal of Physical Chemistry B* **2013**, *117* (35), 10183–10192. https://doi.org/10.1021/jp401512z.
(4) Bergonzo, C.; Hall, K. B.; Cheatham, T. E. Divalent Ion Dependent Conformational Changes in an RNA Stem-Loop Observed by Molecular Dynamics. *J. Chem. Theory Comput.* **2016**, *12* (7), 3382–3389. https://doi.org/10.1021/acs.jctc.6b00173.
(5) Salsbury, A. M.; Lemkul, J. A. Recent Developments in Empirical Atomistic Force Fields for Nucleic Acids and Applications to Studies of Folding and Dynamics. *Current Opinion in Structural Biology* **2021**, *67*, 9–17. https://doi.org/10.1016/j.sbi.2020.08.003.
(6) Zhang, A.; Yu, H.; Liu, C.; Song, C. The Ca2+ Permeation Mechanism of the Ryanodine Receptor Revealed by a Multi-Site Ion Model. *Nat Commun* **2020**, *11* (1), 922. https://doi.org/10.1038/s41467-020-14573-w.
(7) Zuo, Z.; Liu, J. Assessing the Performance of the Nonbonded Mg2+ Models in a Two-Metal-Dependent Ribonuclease. *J. Chem. Inf. Model.* **2019**, *59* (1), 399–408. https://doi.org/10.1021/acs.jcim.8b00627.
(8) Lee, C.; Dommer, A. C.; Schiffer, J. M.; Amaro, R. E.; Grassian, V. H.; Prather, K. A. Cation-Driven Lipopolysaccharide Morphological Changes Impact Heterogeneous Reactions of Nitric Acid with Sea Spray Aerosol Particles. *The Journal of Physical Chemistry Letters* **2021**, *12* (20), 5023–5029. https://doi.org/10.1021/acs.jpclett.1c00810.
(9) Carter-Fenk, K. A.; Dommer, A. C.; Fiamingo, M. E.; Kim, J.; Amaro, R. E.; Allen, H. C. Calcium Bridging Drives Polysaccharide Co-Adsorption to a Proxy Sea Surface Microlayer. *Phys. Chem. Chem. Phys.* **2021**, *23* (30), 16401–16416. https://doi.org/10.1039/D1CP01407B.
(10) Yang, Y.; Jalali, S.; Nilsson, B. L.; Dias, C. L. Binding Mechanisms of Amyloid-like Peptides to Lipid Bilayers and Effects of Divalent Cations. *ACS Chem. Neurosci.* **2021**, *12* (11), 2027–2035. https://doi.org/10.1021/acschemneuro.1c00140.
(11) Li, P.; Roberts, B. P.; Chakravorty, D. K.; Merz, K. M. Rational Design of Particle Mesh Ewald Compatible Lennard-Jones Parameters for +2 Metal Cations in Explicit Solvent. *J. Chem. Theory Comput.* **2013**, *9* (6), 2733–2748. https://doi.org/10.1021/ct400146w.
(12) Mallajosyula, S. S.; Guvench, O.; Hatcher, E.; MacKerell, A. D. CHARMM Additive All-Atom Force Field for Phosphate and Sulfate Linked to Carbohydrates. *J. Chem. Theory Comput.* **2012**, *8* (2), 759–776. https://doi.org/10.1021/ct200792v.
(13) Cochran, R. E.; Laskina, O.; Trueblood, J. V.; Estillore, A. D.; Morris, H. S.; Jayarathne, T.; Sultana, C. M.; Lee, C.; Lin, P.; Laskin, J.; Laskin, A.; Dowling, J. A.; Qin, Z.; Cappa, C. D.; Bertram, T. H.; Tivanski, A. V.; Stone, E. A.; Prather, K. A.; Grassian, V. H. Molecular Diversity of Sea Spray Aerosol Particles: Impact of Ocean Biology on Particle Composition and Hygroscopicity. *Chem* **2017**, *2* (5), 655–667. https://doi.org/10.1016/j.chempr.2017.03.007.
(14) Kirpes, R. M.; Bonanno, D.; May, N. W.; Fraund, M.; Barget, A. J.; Moffet, R. C.; Ault, A. P.; Pratt, K. A. Wintertime Arctic Sea Spray Aerosol Composition Controlled by Sea Ice Lead Microbiology. *ACS Cent Sci* **2019**, *5* (11), 1760–1767. https://doi.org/10.1021/acscentsci.9b00541.
(15) Wang, X.; Sultana, C. M.; Trueblood, J.; Hill, T. C. J.; Malfatti, F.; Lee, C.; Laskina, O.; Moore, K. A.; Beall, C. M.; McCluskey, C. S.; Cornwell, G. C.; Zhou, Y.; Cox, J. L.; Pendergraft, M. A.; Santander, M. V.; Bertram, T. H.; Cappa, C. D.; Azam, F.; DeMott, P. J.; Grassian, V. H.; Prather, K. A. Microbial Control of Sea Spray Aerosol Composition: A Tale of Two Blooms. *ACS Central Science* **2015**, *1* (3), 124–131. https://doi.org/10.1021/acscentsci.5b00148.
(16) Lewis, S. L.; Saliba, G.; Russell, L. M.; Quinn, P. K.; Bates, T. S.; Behrenfeld, M. J. Seasonal Differences in Submicron Marine Aerosol Particle Organic Composition in the North Atlantic. *Frontiers in Marine Science* **2021**, *8*.
(17) Engel, A.; Sperling, M.; Sun, C.; Grosse, J.; Friedrichs, G. Organic Matter in the Surface Microlayer: Insights From a Wind Wave Channel Experiment. *Frontiers in Marine Science* **2018**, *5*.
(18) Triesch, N.; van Pinxteren, M.; Salter, M.; Stolle, C.; Pereira, R.; Zieger, P.; Herrmann, H. Sea Spray Aerosol Chamber Study on Selective Transfer and Enrichment of Free and Combined Amino Acids. *ACS Earth Space Chem.* **2021**, *5* (6), 1564–1574. https://doi.org/10.1021/acsearthspacechem.1c00080.

(19) Santander, M. V.; Schiffer, J. M.; Lee, C.; Axson, J. L.; Tauber, M. J.; Prather, K. A. Factors Controlling the Transfer of Biogenic Organic Species from Seawater to Sea Spray Aerosol. *Sci Rep* **2022**, *12* (1), 3580. https://doi.org/10.1038/s41598-022-07335-9.

(20) Triesch, N.; van Pinxteren, M.; Frka, S.; Stolle, C.; Spranger, T.; Hoffmann, E. H.; Gong, X.; Wex, H.; Schulz-Bull, D.; Gašparović, B.; Herrmann, H. Concerted Measurements of Lipids in Seawater and on Submicrometer Aerosol Particles at the Cabo Verde Islands: Biogenic Sources, Selective Transfer and High Enrichments. *Atmospheric Chemistry and Physics* **2021**, *21* (6), 4267–4283. https://doi.org/10.5194/acp-21-4267-2021.

(21) Cochran, R. E.; Laskina, O.; Jayarathne, T.; Laskin, A.; Laskin, J.; Lin, P.; Sultana, C.; Lee, C.; Moore, K. A.; Cappa, C. D.; Bertram, T. H.; Prather, K. A.; Grassian, V. H.; Stone, E. A. Analysis of Organic Anionic Surfactants in Fine and Coarse Fractions of Freshly Emitted Sea Spray Aerosol. *Environmental Science and Technology* **2016**, *50* (5), 2477–2486. https://doi.org/10.1021/acs.est.5b04053.

(22) Malfatti, F.; Lee, C.; Tinta, T.; Pendergraft, M. A.; Celussi, M.; Zhou, Y.; Sultana, C. M.; Rotter, A.; Axson, J. L.; Collins, D. B.; Santander, M. V.; Anides Morales, A.; Aluwihare, L. I.; Riemer, N.; Grassian, V. H.; Azam, F.; Prather, K. A. Detection of Active Microbial Enzymes in Nascent Sea Spray Aerosol: Implications for Atmospheric Chemistry and Climate. *Environmental Science & Technology Letters* **2019**. https://doi.org/10.1021/acs.estlett.8b00699.

(23) Barbe, S.; Lafaquière, V.; Guieysse, D.; Monsan, P.; Remaud-Siméon, M.; André, I. Insights into Lid Movements of Burkholderia Cepacia Lipase Inferred from Molecular Dynamics Simulations. *Proteins: Structure, Function, and Bioinformatics* **2009**, *77* (3), 509–523. https://doi.org/10.1002/prot.22462.

(24) Luo, M.; Dommer, A. C.; Schiffer, J. M.; Rez, D. J.; Mitchell, A. R.; Amaro, R. E.; Grassian, V. H. Surfactant Charge Modulates Structure and Stability of Lipase-Embedded Monolayers at Marine-Relevant Aerosol Surfaces. *Langmuir* **2019**, *35* (27), 9050–9060. https://doi.org/10.1021/acs.langmuir.9b00689.

(25) Pires De Oliveira, I.; Jara, G. E. Molecular Mechanism of Activation of Burkholderia Cepacia Lipase at Aqueous-Organic Interfaces. *Phys. Chem. Chem. Phys* **2017**, *19*, 31499–31499. https://doi.org/10.1039/c7cp04466f.

(26) Schiffer, J. M.; Luo, M.; Dommer, A. C.; Thoron, G.; Pendergraft, M.; Santander, M. V.; Lucero, D.; Pecora De Barros, E.; Prather, K. A.; Grassian, V. H.; Amaro, R. E. Impacts of Lipase Enzyme on the Surface Properties of Marine Aerosols. *J. Phys. Chem. Lett* **2018**, *9*, 22–22. https://doi.org/10.1021/acs.jpclett.8b01363.

(27) Sánchez, D. A.; Tonetto, G. M.; Ferreira, M. L. Burkholderia Cepacia Lipase: A Versatile Catalyst in Synthesis Reactions. *Biotechnology and Bioengineering* **2018**, *115* (1), 6–24. https://doi.org/10.1002/bit.26458.

(28) Angle, K. J.; Crocker, D. R.; Simpson, R. M. C.; Mayer, K. J.; Garofalo, L. A.; Moore, A. N.; Garcia, S. L. M.; Or, V. W.; Srinivasan, S.; Farhan, M.; Sauer, J. S.; Lee, C.; Pothier, M. A.; Farmer, D. K.; Martz, T. R.; Bertram, T. H.; Cappa, C. D.; Prather, K. A.; Grassian, V. H. Acidity across the Interface from the Ocean Surface to Sea Spray Aerosol. *PNAS* **2021**, *118* (2). https://doi.org/10.1073/pnas.2018397118.

(29) Luo, M.; Wauer, N. A.; Angle, K. J.; Dommer, A. C.; Song, M.; Nowak, C. M.; Amaro, R. E.; Grassian, V. H. Insights into the Behavior of Nonanoic Acid and Its Conjugate Base at the Air/Water Interface through a Combined Experimental and Theoretical Approach. *Chemical Science* **2020**, *11* (39). https://doi.org/10.1039/d0sc02354j.

(30) Wellen, B. A.; Lach, E. A.; Allen, H. C. Surface p: K a of Octanoic, Nonanoic, and Decanoic Fatty Acids at the Air-Water Interface: Applications to Atmospheric Aerosol Chemistry. *Physical Chemistry Chemical Physics* **2017**, *19* (39), 26551–26558. https://doi.org/10.1039/c7cp04527a.

(31) Budin, I.; Prwyes, N.; Zhang, N.; Szostak, J. W. Chain-Length Heterogeneity Allows for the Assembly of Fatty Acid Vesicles in Dilute Solutions. *Biophysical Journal* **2014**, *107* (7), 1582–1590. https://doi.org/10.1016/j.bpj.2014.07.067.

(32) Budin, I.; Debnath, A.; Szostak, J. W. Concentration-Driven Growth of Model Protocell Membranes. *Journal of the American Chemical Society* **2012**, *134* (51), 20812–20819. https://doi.org/10.1021/ja310382d.

(33) Luo, M. Structure, Properties, Dynamics, and Photochemical Behavior of Organic and Biological Species in Marine-Relevant Environments. PhD Thesis, 2021. https://www.proquest.com/dissertations-theses/structure-properties-dynamics-photochemical/docview/2511901748/se-2.

(34) Theodorou, D. N.; Suter, U. W. Shape of Unperturbed Linear Polymers: Polypropylene. *Macromolecules* **1985**, *18* (6), 1206–1214. https://doi.org/10.1021/ma00148a028.

(35) Humphery Dalke, A. S., K. ,. W. VMD - Visual Molecular Dynamics. *J. Molec. Graph* **1996**, *14*, 33–38.

(36) Karadima, K. S.; Mavrantzas, V. G.; Pandis, S. N. Insights into the Morphology of Multicomponent Organic and Inorganic Aerosols from Molecular Dynamics Simulations. *Atmospheric Chemistry and Physics* **2019**, *19* (8), 5571–5587. https://doi.org/10.5194/acp-19-5571-2019.

(37) Gowers, R. J.; Linke, M.; Barnoud, J.; Reddy, T. J. E.; Melo, M. N.; Seyler, S. L.; Domański, J.; Dotson, D. L.; Buchoux, S.; Kenney, I. M.; Beckstein, O. MDAnalysis: A Python Package for the Rapid Analysis of Molecular Dynamics Simulations. In *Proceedings of the 15th Python in Science Conference*; Benthall, S., Rostrup, S., Eds.; 2016; pp 98–105. https://doi.org/10.25080/Majora-629e541a-00e.

(38) Calandrini, V.; Pellegrini, E.; Calligari, P.; Hinsen, K.; Kneller, G. R. NMoldyn - Interfacing Spectroscopic Experiments, Molecular Dynamics Simulations and Models for Time Correlation Functions. *JDN* **2011**, *12*, 201–232. https://doi.org/10.1051/sfn/201112010.

(39) Buyl, P. de. Tidynamics: A Tiny Package to Compute the Dynamics of Stochastic and Molecular Simulations. *Journal of Open Source Software* **2018**, *3* (28), 877. https://doi.org/10.21105/joss.00877.

(40) Maginn, E. J.; Messerly, R. A.; Carlson, D. J.; Roe, D. R.; Elliot, J. R. Best Practices for Computing Transport Properties 1. Self-Diffusivity and Viscosity from Equilibrium Molecular Dynamics [Article v1.0]. *Living Journal of Computational Molecular Science* **2019**, *1* (1), 6324–6324. https://doi.org/10.33011/livecoms.1.1.6324.

(41) Holz, M.; Heil, S. R.; Sacco, A. Temperature-Dependent Self-Diffusion Coefficients of Water and Six Selected Molecular Liquids for Calibration in Accurate 1H NMR PFG Measurements. *Phys. Chem. Chem. Phys.* **2000**, *2* (20), 4740–4742. https://doi.org/10.1039/B005319H.

(42) McGibbon, R. T.; Beauchamp, K. A.; Harrigan, M. P.; Klein, C.; Swails, J. M.; Hernández, C. X.; Schwantes, C. R.; Wang, L.-P.; Lane, T. J.; Pande, V. S. MDTraj: A Modern Open Library for the Analysis of Molecular Dynamics Trajectories. *Biophysical Journal* **2015**, *109* (8), 1528–1532. https://doi.org/10.1016/j.bpj.2015.08.015.

(43) Harris, C. R.; Millman, K. J.; van der Walt, S. J.; Gommers, R.; Virtanen, P.; Cournapeau, D.; Wieser, E.; Taylor, J.; Berg, S.; Smith, N. J.; Kern, R.; Picus, M.; Hoyer, S.; van Kerkwijk, M. H.; Brett, M.; Haldane, A.; del Río, J. F.; Wiebe, M.; Peterson, P.; Gérard-Marchant, P.; Sheppard, K.; Reddy, T.; Weckesser, W.; Abbasi, H.; Gohlke, C.; Oliphant, T. E. Array Programming with NumPy. *Nature* **2020**, *585* (7825), 357–362. https://doi.org/10.1038/s41586-020-2649-2.

(44) Klamkin, M. S. Elementary Approximations to the Area of N-Dimensional Ellipsoids. *The American Mathematical Monthly* **1971**, *78* (3), 280–283. https://doi.org/10.2307/2317530.

(45) Dunkl, C. F.; Ramirez, D. E. Computing Hyperelliptic Integrals for Surface Measure of Ellipsoids. *ACM Trans. Math. Softw.* **1994**, *20* (4), 413–426. https://doi.org/10.1145/198429.198430.

(46) Dunkl, C. F.; Ramirez, D. E. Algorithm 736: Hyperelliptic Integrals and the Surface Measure of Ellipsoids. *ACM Trans. Math. Softw.* **1994**, *20* (4), 427–435. https://doi.org/10.1145/198429.198431.

(47) Douglas A. Skoog; F. James Holler; Stanley R. Crouch. *Principles of Instrumental Analysis*, 6th ed.; Thomson Brooks/Cole, 2007.
