# Supplementary material for: Revealing the Impacts of Chemical Complexity on Submicrometer Sea Spray Aerosol Morphology
Source: ACS Cent Sci. 2023 May 4;9(6):1088–103. doi: 10.1021/acscentsci.3c00184 (PMC10311664; doi:10.1021/acscentsci.3c00184)
Supplement: Supplementary file 7 — oc3c00184_si_008.pdf [file oc3c00184_si_008.pdf]

oc-2023-00184d.R1

Name: Peer Review Information for "Revealing the Impacts of Chemical Complexity on Submicron Sea Spray Aerosol Morphology"

First Round of Reviewer Comments

Reviewer: 1

Comments to the Author

This manuscripts describes a molecular dynamics computational investigation of the structure of simulated sea spray aerosol. The particle compositions explored represent the chemical complexity of nascent SSA while maintaining some necessary simplifications to ensure the MD methods are accurate. The authors explore the phase morphology and distribution of chemical species across the bulk to the surface, with fascinating results that give a unique insight into the microscopic world of aerosol particles. These insights are not possible with experimental methods and previous computational studies have been limited in size. That the authors were able to simulate particles of a relevant size range with the level of detail described is an incredible achievement. Overall, this is a wonderfully written paper on a very interesting topic using truly novel and impactful techniques. It was a pleasure to read and I recommend publication as-is.

Reviewer: 2

Comments to the Author

Review of “Revealing the impacts of chemical complexity on submicron sea spray aerosol morphology” by Dommer et al.

The study uses molecular dynamics simulations to investigate the morphology of sea spray aerosol (SSA) proxies. The results provide unprecedented detail into the structure of these proxies. The paper is well-written, and the figures are fascinating.

Overall, the results show that the surfactants in sea spray aerosol likely do not form a tightly packed shell at the surface of the aerosol particles. These results are important since a tightly packed shell could inhibit the kinetics of water uptake and heterogeneous reactions.

Below are specific comments that the authors should address adequately before publication.

1) Abstract, page 1, lines 48-53: “our work shows that morphological mechanisms underlying why submicron SSA readily absorb water – and thus have a higher cloud forming potential-than would otherwise be predicted for organic-rich aerosol.” I think this is too general of a statement and may be misleading to some. The authors should more specifically indicate what they have shown. E.g., the

authors have shown that surfactant molecules do not likely inhibit the kinetic uptake of water on the SSA since the surfactants do not form a tightly packed layer. The original statement makes it sound like the morphology impacts the equilibrium water content, which I don't think the authors have shown in the current manuscript. Also, "organic-rich aerosol" is too vague.

2) Page 17, line 52. "FAs may thus impede the diffusion of water by confining the water to smaller volumetric regions inside the particle, imposing physical membrane-like barriers." Does this statement contradict the statement in the abstract that states that "SSAs readily absorb water"?

3) The authors studied 40 nm aerosol particles, smaller than most sea spray aerosol (SSA). Can they safely extrapolate to larger sizes?

4) Page 1, lines 28-30. "yet observations indicate that these aerosols readily uptake water as if they were salt particles." Are the authors implying that the particles have the same hygroscopic properties (i.e., kappa value) as salt particles, or are they suggesting that there do not appear to be any kinetic limitations to water uptake? Please clarify.

5) Page 11, line 53: "FAs, particularly protonated FAs, have high hydrophobicity and favor formation of highly ordered structures". Do fatty acids ionize in sea spray aerosols? If so, will the ionization of the head groups in the fatty acids affect your results? Please discuss.

6) Please indicate the ratio of organic to inorganic material used in the simulations. Sorry if I missed this point.

7) Results, Page 11, lines 24-24: "Equilibrium was assumed to be reached after approximately 100 ns of production". Do the authors think the structures would be the same if they could run the simulations for minutes to hours (similar to real-world conditions)? Please discuss.

8) I do not entirely follow what the authors show in Figure 7C-D. On page 22, the authors state that bright regions of the images indicate high organic content, while dark regions indicate interfacial water. In Figure C, I see white streaks, and I think the authors are assigning these streaks to LPS. Could they also be regions where the monolayer has exceeded the equilibrium spreading pressure and collapsed/buckled and formed regions with multiple layers? For D, I only see large black regions on a white background. Are these features "holes"? Is there anything else I should take away from D?

9) Page 24, lines 11-14. Here, and in other places in the document, I was unsure if the authors were implying that morphology influences the equilibrium water content or water uptake kinetics. I think the results are only related to kinetics. Regardless, please clarify what you mean by "high hygroscopicity."

10) For the BAM results, what was the surface pressure for the images shown in Figure 7C-D? How do the images change with surface pressure? These images may provide additional insight, and the authors should consider adding them to the SI. I think the BAM section needs some improvement.

Author's Response to Peer Review Comments:

AFFILIATIONS: Please ensure that the full details of author affiliations are listed in the manuscript file and Supporting Information file. The following details are required: institution, city, postal code (if existing), and country.

Postal codes and country added.

CORR AU EMAIL: Please label the corresponding author's email address as "email" in the manuscript and Supporting Information file.

Done.

ABSTRACT: Please shorten the abstract to 200 words or less.

Abstract shortened to 200 words.

REFERENCES: Please include author names, article titles, journal name, publication year, and at least the first page for each reference citation for the following incomplete journal references: 4, 5, 54, 55, 56, 60, 61, 62, 63, 64, 65, 94, 97, 99, 143.

Completed.

SYNOPSIS: ACS Central Science requires a brief synopsis. The synopsis should be no more than 200 characters (including spaces) and should reasonably correlate with the Table of Contents (TOC) graphic. The synopsis is intended to explain the importance of the article to a broader readership across the sciences. Please place your synopsis in the manuscript file after the TOC graphic.

Synopsis text: Increased chemical complexity in submicron sea spray aerosol leads to more heterogeneous morphology and lower surfactant coverage, which may explain high hygroscopicity of nascent organic-rich SSA.

SI STATEMENT: Because your manuscript is accompanied by Supporting Information for publication, a brief description of the supplementary material is required in the manuscript. The appropriate format is: Supporting Information (header), followed by a brief statement in nonsentence format listing the contents of the material supplied as Supporting Information.

Section added to the manuscript file.

SI PAGINATION: SI pages must be numbered consecutively, starting with page S1.

Done.

SI LINE-NUMBERING: Please remove the line-numbering from your Supporting Information file.

Line-numbering removed.

SI MOVIE CAPTIONS: Please add the list of captions for your SI movies in your main Supporting Information file and in the Supporting Information paragraph in the manuscript. These captions should not be uploaded as a separate file.

Brief captions added to the manuscript file.

Reviewer(s)' Comments to Author:

Reviewer: 1
Recommendation: Publish in ACS Central Science without change.
Comments:
This manuscript describes a molecular dynamics computational investigation of the structure of simulated sea spray aerosol. The particle compositions explored represent the chemical complexity of nascent SSA while maintaining some necessary simplifications to ensure the MD methods are accurate. The authors explore the phase morphology and distribution of chemical species across the bulk to the surface, with fascinating results that give a unique insight into the microscopic world of aerosol particles. These insights are not possible with experimental methods and previous computational studies have been limited in size. That the authors were able to simulate particles of a relevant size range with the level of detail described is an incredible achievement. Overall, this is a wonderfully written paper on a very interesting topic using truly novel and impactful techniques. It was a pleasure to read and I recommend publication as-is.
Additional Questions:
Quality of experimental data, technical rigor: Top 1%
Significance to chemistry researchers in this and related fields: Top 1%
Broad interest to other researchers: Top 1%
Novelty: Top 1%
Is this research study suitable for media coverage or a First Reactions (a News & Views piece in the journal)?: Yes

----

Reviewer: 2
Recommendation: Publish in ACS Central Science after minor revisions noted.
Comments:
Review of "Revealing the impacts of chemical complexity on submicron sea spray aerosol morphology" by Dommer et al.
The study uses molecular dynamics simulations to investigate the morphology of sea spray aerosol (SSA) proxies. The results provide unprecedented detail into the structure of these proxies. The paper is well-written, and the figures are fascinating.
Overall, the results show that the surfactants in sea spray aerosol likely do not form a tightly packed shell at the surface of the aerosol particles. These results are important since a tightly packed shell could inhibit the kinetics of water uptake and heterogeneous reactions.
Below are specific comments that the authors should address adequately before publication.

1) Abstract, page 1, lines 48-53: "our work shows that morphological mechanisms underlying why submicron SSA readily absorb water – and thus have a higher cloud forming potential-than would otherwise be predicted for organic-rich aerosol." I think this is too general of a statement and may be misleading to some. The authors should more specifically indicate what they have shown. E.g., the authors have shown that surfactant molecules do not likely inhibit the kinetic uptake of water on the SSA since the surfactants do not form a tightly packed layer. The original statement makes it sound like the morphology impacts the equilibrium water content, which I don't think the authors have shown in the current manuscript. Also, "organic-rich aerosol" is too vague.

We thank the reviewer for this insight! We have changed this line in the abstract to read: "These observations indicate that increased chemical complexity in submicron SSA leads to a reduced surface coverage by marine organics, which can facilitate water uptake in the atmosphere and particle hygroscopicity. Our work thus establishes large-scale MD simulations as a novel technique for interrogating aerosols at the single-particle level."

This change clarifies specifically what we found in the study and its direct impacts.

2) Page 17, line 52. "FAs may thus impede the diffusion of water by confining the water to smaller volumetric regions inside the particle, imposing physical membrane-like barriers." Does this statement contradict the statement in the abstract that states that "SSAs readily absorb water"?

We thank the reviewer for this question! We do not believe that these statements are contradictory. We hypothesize that the particle surface represents the kinetic barrier to water uptake, and that the distribution of organics in the aqueous phase inhibits the diffusion of water throughout the particle. As the particle uptakes more and more water, however, the arrangement of organics is likely to change in the process which may serve to dissolve portions of the membrane-like barriers.

3) The authors studied 40 nm aerosol particles, smaller than most sea spray aerosol (SSA). Can they safely extrapolate to larger sizes?

We thank the reviewer for this great question. To address it, we have added the following discussion to the main text ("Extrapolating to Longer Time and Length Scales") as we believe it is an important point that was not clearly outlined in our original manuscript (and as well, it brings in new references):

Extrapolating to Longer Time and Length Scales

Regarding the extrapolation of our findings to longer time and length scales, we believe that these results can be safely extrapolated to SSA diameters up to 200 nm, which have the same or similar organic content. For submicron SSA, specifically those <200 nm in diameter, the organic:inorganic mass ratio is > 4:1 and for even smaller particles, this ratio can increase to ~44:1.[16,78] This mass ratio is primarily governed by the production mechanism of the particle. As SSA diameter increases above 200 nm, the ratio changes dramatically towards higher inorganic content (reaching >80% inorganics) due to a shift in the dominant SSA production mechanism for larger particle sizes. For example, submicron SSA, like those in our study, are traditionally associated with film drops and contain more water-insoluble material, while jet drops produce supermicron particles and tend to contain more inorganic species.[93]

The high organic content of submicron SSA produced via bursting film caps, as well as the diversity in chemical properties of the organic content, lead to the unique morphologies we see in this study. We expect that a moderate increase in diameter, up to ~200 nm, would lead to only subtle differences in the interfacial and bulk morphologies. This size increase would cause 1) a decrease in the particle curvature; and 2) a decrease in the surface area-to-volume ratio. A decreased curvature caused by water uptake has been shown experimentally to facilitate the growth of surfactant lipid domains on microdroplets.[142] The tessellated rafts seen in our simulations would likely increase in size, but would remain as separate domains. Furthermore, BAM images indicate that we would still expect the same interfacial morphologies with respect to LPS and BCL as the surface curvature approaches zero.

Increasing particle size would also decrease the surface area-to-volume ratio, yet this would not result in significant morphological or rheological differences assuming all other factors remain constant. The FAs would still self-assemble into amorphous oil droplets and bisecting bilayer structures, which is characteristic for FAs at such low pH.[143–145] FA vesicles are also formed at low pH in the aqueous phase; however, FAs tend to form vesicles with diameters ranging from 50 nm – 1.5 μm.[146,147] With additional organics disrupting the highly ordered surfactants, it is possible that full vesicle formation could also occur in submicron SSA. Additionally, LPS is known to self-assemble into bilayer structures in the

aqueous phase, but the thickness of one LPS bilayer approaches 200 nm; it is thus more likely that LPS will exhibit similar properties to those in the present study.

As time scales are extended, the overall morphology of these structures is expected to remain consistent. FA aggregation occurs rapidly (<50 ns) as demonstrated in SI Movie M1, with aggregates stabilized by strong hydrophobic interactions between tail groups. While we do not predict major structural differences to arise at extended times, small differences such as the reorganization of lipids within the aggregates themselves are likely to occur. This process is much slower, however, and unlikely to be observed via all-atom MD. For example, experimental and theoretical studies show that lipid raft formation in biological membranes occurs on the order of micro- to milliseconds.[148,149]

In addition, these simulations are representative models for nascent SSA which are consequentially linked only to the early lifetime of these particles. Experimental work has shown that as nascent SSA age, their morphology drastically changes with exposure to gas phase oxidants such as OH radicals and ozone.[150] Because these particles exist in a dynamic environment where they constantly undergo compositional changes, a simulation of these particles for minutes to hours would not accurately represent the real life-cycle of nascent SSA. Thus, the simulations presented in this study represent the important metastable states that nascent SSA develop during their early lifetime.

4) Page 1, lines 28-30. "yet observations indicate that these aerosols readily uptake water as if they were salt particles." Are the authors implying that the particles have the same hygroscopic properties (i.e., kappa value) as salt particles, or are they suggesting that there do not appear to be any kinetic limitations to water uptake? Please clarify.

We thank the reviewer for this question! Yes, according to a variety of experimental observations, hygroscopicity parameters (κ) for submicron sea spray are measured within the range of 0.8-1.2, with an average value of 0.95, which is closer to the range of pure salty particles ($\kappa > 1.0$) than that of pure organics. According to the linear mixing rule for determining hygroscopicity parameters, the volume fraction of the organic material can be derived to be <0.4, which is inconsistent with the actual, much higher, volume fractions, which is the discrepancy we wanted to address in the manuscript.

In order to clarify this point, we have adjusted the second paragraph of the introduction to read:

"Fine SSA in particular, with diameters <200 nm, contain nearly 100% organic material by mass, yet observations indicate that these aerosols have hygroscopicity parameters approaching those of pure salt particles, i.e., they uptake water as if they were salty."

5) Page 11, line 53: "FAs, particularly protonated FAs, have high hydrophobicity and favor formation of highly ordered structures". Do fatty acids ionize in sea spray aerosols? If so, will the ionization of the head groups in the fatty acids affect your results? Please discuss.

We thank the reviewer for this question. In the extended rationale for the experimental design provided in the SI, we discuss the decision of including only fully protonated FAs. SSAs rapidly acidify upon release into the atmosphere due to the dissolution of acidic gasses and rapid dehydration which results in an increased concentration of acidic compounds. Angle et al. found that freshly emitted submicron SSAs had a pH of ~2. Due to this we expect nearly all FAs to remain fully protonated, as depicted in our model.[9]

6) Please indicate the ratio of organic to inorganic material used in the simulations. Sorry if I missed this point.

We thank the reviewer for this question and realize that it was not explicitly addressed in the main text. The ratio of inorganic : organic : water mass is 0.09 : 0.40 : 0.51, and it has been added to the second paragraph of the experimental design under the methods section.

7) Results, Page 11, lines 24-24: "Equilibrium was assumed to be reached after approximately 100 ns of production". Do the authors think the structures would be the same if they could run the simulations for minutes to hours (similar to real-world conditions)? Please discuss.

We thank the reviewer for this question. We expect that the general structures would remain consistent as diffusion and insolubility of the FAs in water are the driving forces of their formation, which is a rapid process that occurs on very short timescales (<50ns) as demonstrated in the rapid ordering of the FAs in Movie M1. Major structural differences are unlikely to arise, but small differences such as the reorganization of lipids within the aggregates themselves may occur. This process is much slower and, as seen in other simulations of lipids, e.g., lipid raft formation, would require timescales of micro- to milliseconds.[10,11] Additionally, these simulations are representative models for nascent SSA which are consequentially linked only to the early lifetime of these particles. Experimental work has shown that as nascent SSA age, their morphology drastically changes.[12] Because these particles exist in a dynamic environment where they constantly undergo compositional changes, a simulation of these particles for minutes to hours would not accurately represent the real life-cycle of nascent SSA. Thus, the simulations presented in this study represent the important metastable states that nascent SSA develop during their early lifetime.

The above points are now discussed in the new section entitled, "Extrapolating to Longer Time and Length Scales", as discussed above.

8) I do not entirely follow what the authors show in Figure 7C-D. On page 22, the authors state that bright regions of the images indicate high organic content, while dark regions indicate interfacial water. In Figure C, I see white streaks, and I think the authors are assigning these streaks to LPS. Could they also be regions where the monolayer has exceeded the equilibrium spreading pressure and collapsed/buckled and formed regions with multiple layers? For D, I only see large black regions on a white background. Are these features "holes"? Is there anything else I should take away from D?

We thank the reviewer for these questions. We have now explained further in the main text the bright and dark regions. In addition, according to the isotherms, which are now provided in the SI, we have not exceeded the equilibrium spreading pressure. We have clarified that we interpret the white streaks as primarily fatty acids. The reviewer has correctly identified the black regions as "holes" which have lower fatty acid content than their surroundings. A potential explanation is that BCL forms clusters and inserts into the monolayer at these locations that we see as holes; subsequently, the BCL aggregates sink into the aqueous phase due to the decreased surface affinity of the cluster compared to the single BCL units. (This hypothesis is discussed in the paragraph beginning with "BCL (Figure 7D)..."

The overall takeaway for Figure C vs. D is that LPS and BCL are experimentally observed to cause dramatic variations in interfacial morphology, consistent with computational results (albeit at a different scale due to technical limitations).

9) Page 24, lines 11-14. Here, and in other places in the document, I was unsure if the authors were implying that morphology influences the equilibrium water content or water uptake kinetics. I think the results are only related to kinetics. Regardless, please clarify what you mean by "high hygroscopicity."

We thank the reviewer for their comment. We have clarified that we are referring to kinetics by adding a citation to experimental results showing that water evaporation rates are impacted by heterogeneous monolayers (including experiments with LPS). By "high hygroscopicity," we refer to the following:

1) SSA do uptake considerable quantities of water (sometimes modeled by the hygroscopicity of NaCl);
2) Long chain saturated fatty acids form tightly-packed monolayers that are impermeable to water;

3) If SSA exteriors were fully coated by tightly packed fatty acids as previously suggested, we would expect very low water uptake and hence low experimental hygroscopicity. Since hygroscopicity is in fact observed to be high, something with the traditional view is inconsistent. Our observation that nascent SSA interfaces are highly heterogeneous resolves this inconsistency. We have added a sentence to the end of the stated paragraph to succinctly convey this idea.

10) For the BAM results, what was the surface pressure for the images shown in Figure 7C-D? How do the images change with surface pressure? These images may provide additional insight, and the authors should consider adding them to the SI. I think the BAM section needs some improvement.

We thank the reviewer for these questions and comments! The surface pressure for the images shown in Figure 7C-D was ~30 mN/m. We have provided the additional data, including images at different surface pressures in the SI and have expanded the discussion of BAM as it pertains to these systems.
